# TERRA G-quadruplex RNA interaction with TRF2 GAR domain is required for telomere integrity

**DOI:** 10.1038/s41598-021-82406-x

**Published:** 2021-02-10

**Authors:** Yang Mei, Zhong Deng, Olga Vladimirova, Nitish Gulve, F. Brad Johnson, William C. Drosopoulos, Carl L. Schildkraut, Paul M. Lieberman

**Affiliations:** 1 The Wistar Institute, Philadelphia, PA 19104 USA; 2 Department of Pathology, Perelman School of Medicine at the University of Pennsylvania, Philadelphia, PA 19104 USA; 3 Department of Cell Biology, Albert Einstein College of Medicine, 1300 Morris Park Avenue, The Bronx, NY 10461 USA

**Keywords:** Telomeres, Long non-coding RNAs

## Abstract

Telomere dysfunction causes chromosomal instability which is associated with many cancers and age-related diseases. The non-coding telomeric repeat-containing RNA (TERRA) forms a structural and regulatory component of the telomere that is implicated in telomere maintenance and chromosomal end protection. The basic N-terminal Gly/Arg-rich (GAR) domain of telomeric repeat-binding factor 2 (TRF2) can bind TERRA but the structural basis and significance of this interaction remains poorly understood. Here, we show that TRF2 GAR recognizes G-quadruplex features of TERRA. We show that small molecules that disrupt the TERRA-TRF2 GAR complex, such as N-methyl mesoporphyrin IX (NMM) or genetic deletion of TRF2 GAR domain, result in the loss of TERRA, and the induction of γH2AX-associated telomeric DNA damage associated with decreased telomere length, and increased telomere aberrations, including telomere fragility. Taken together, our data indicates that the G-quadruplex structure of TERRA is an important recognition element for TRF2 GAR domain and this interaction between TRF2 GAR and TERRA is essential to maintain telomere stability.

## Introduction

Telomeres are nucleoprotein structures that protect and maintain the ends of linear chromosomes and are necessary for genomic stability^1–4^. Telomeres consist of variable length repetitive DNA (typically 5′-TTAGGG-3′) bound by a group of six essential proteins termed shelterin^5–7^. The TTAGGG sequence is recognized by two sequence-specific myb-family DNA binding shelterin proteins termed TRF1 and TRF2^8–10^. Telomeric DNAs terminate with a single-stranded G-rich 3′-overhang of TTAGGG repeats^11^. TRF2 facilitates the invasion of this single-stranded overhang into the telomeric double-stranded DNA to form a structure termed a T-loop which protects against DNA damage^12–14^.

Telomere repeat DNA can be transcribed to generate telomeric repeat-containing RNAs (TERRA)^15,16^. TERRA are expressed at variable levels from different chromosomes and subject to regulation by cell stress and DNA damage signals, as well as cell cycle and developmental states^17,18^. TERRA has been shown to be involved in numerous functions including regulation of telomerase activity, inhibition of histone methyltransferase LSD1, competition with single-stranded DNA-binding proteins, and modulation of telomeric chromatin^19–25^. Several mechanisms have been identified for these functions of TERRA, including direct interaction with telomeric proteins and formation of RNA–DNA hybrids that regulate the access of DNA polymerase or telomerase^26–31^.

The G-rich TERRAs, as well as the telomeric DNAs, are known to form G-quadruplexes (G4) which contain stacked Hoogsteen-bonded G-quartet motifs stabilized by monovalent cation such as K^+^ and Na^+^^32–34^. The formation of telomeric G4 has been shown to inhibit telomerase activity^35,36^, making it a potential target for anti-cancer drug design. Structural studies have shown that the stacking interaction of planar G-quartets is important for ligand targeting^37–41^ and higher order arrangement of G4 have been observed in long TERRA sequences^32,42^. Several telomeric DNA G4 specific binding compounds have been developed^43–45^. Among them, BRACO-19 was shown to bind telomeric G4 DNA, induce telomere DNA damage, and inhibit telomerase activity and cell proliferation in human cancer cells^46–51^. The water-soluble N-methyl mesoporphyrin IX (NMM) was also shown to bind telomeric DNA G4 by X-ray crystallography though with low binding affinity^52^. NMM shows high selectivity for G4 DNA over other DNA structures^53–55^. It also prefers parallel G4 folds over antiparallel folds^54,56,57^. Like BRACO-19, NMM was shown to inhibit telomerase activity, making it another good candidate for cancer treatment^58^. TERRA can also form G4 RNA structures, but it is not yet known how these G4 interacting molecules affect TERRA regulation and function.

We and others have shown that TERRA can interact directly with the glycine-arginine rich (GAR) element (also referred to as RGG) in the TRF2 amino terminal basic domain^59–63^. Biophysical studies have found that the G4 structure formation of TERRA is required for TRF2 binding^64,65^. Our previous research indicated that TERRA interaction with TRF2 GAR was important for telomeric heterochromatin formation, ORC recruitment, and telomere DNA integrity^61^. However, the TRF2 GAR domain can also bind to telomere DNA structures, including 4-way junctions formed at telomere T-loops^60,66^. Here, we set out to investigate the ability of G4 interacting molecules, such as BRACO-19 and NMM, to bind TERRA and disrupt its interaction with the TRF2 GAR domain. We also assayed their effects on TERRA expression and telomeric DNA integrity in living cells, and whether these effects resemble the effects of genetic disruption of the TRF2 GAR domain.

## Results

### NMM preferentially binds TERRA RNA compared to telomere G4 DNA

**Figure 1:**
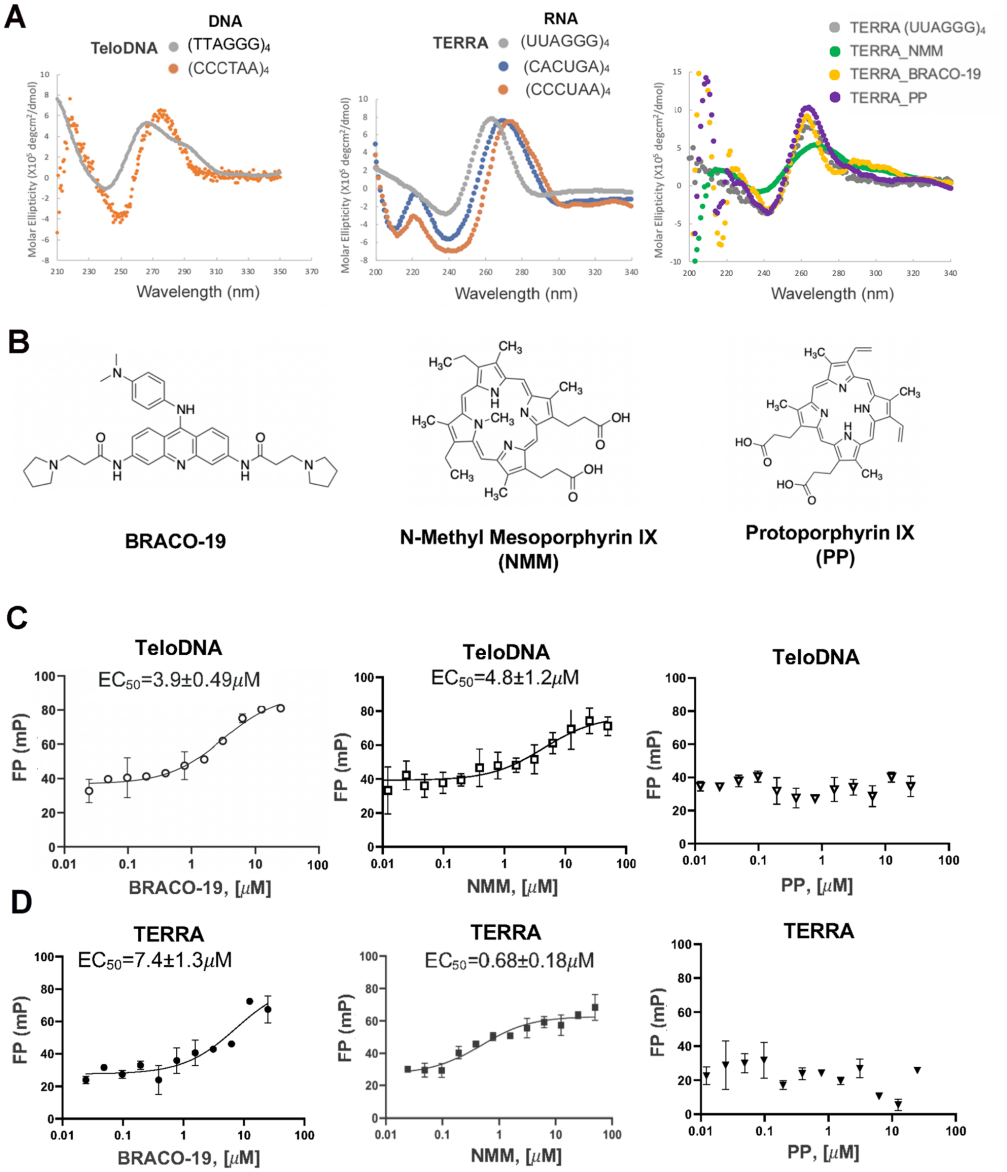
G4 features of TERRA and TeloDNA oligos correlate with binding to G4-interacting compounds. (**A**) CD spectroscopy results for different oligos in 150 mM KCl buffer. Left panel: TeloDNA (grey), antisense DNA (orange); middle panel: TERRA (UUAGGG)_4_ (grey), or RNA controls (CACUGA)_4_ (blue) and (CCCUAA)_4_ (orange); right panel: TERRA (grey), TERRA mixed with NMM (green), BRACO-19 (yellow), and PP (purple) by 1:1 molar ratio. (**B**) Chemical structure of BRACO-19, N-Methyl Mesoporphyrin (NMM) and Protoporphyrin IX (PP). (**C–D**) FP assays for 5′ fluorescein-labeled TeloDNA (**C**) and TERRA (**D**) probes (10 nM) were mixed with BRACO-19, NMM and PP, respectively over the range of concentrations shown. All resulting FP values were measured from 3 independent experiments and plotted as a function of compound concentration using Prism 8.0. EC_50_ were presented in each graph.

Both TERRA and telomere repeat DNA are known to form distinct G4 structures. We synthesized 24mer versions of TERRA and telomeric G-rich strand DNA (TeloDNA) containing the minimal 4 repeats necessary to form respective G4 structures. Circular dichroism confirmed that both the 24mer TeloDNA and TERRA formed G4 structures while the 24mer antisense TeloDNA or TERRA mutants failed to form a G4 (Fig. 1A). TeloDNA and TERRA showed typical secondary structure features of G-quadruplex, with a positive peak at 260 nm and a negative peak at 240 nm. Antisense DNA profile showed a typical single-stranded DNA with a positive peak at around 280 nm and a negative peak at about 250 nm (Fig. 1A, left panel). CD profiles of the two control RNAs showed typical secondary structure features of a random RNA with right shifted positive peak compared to G-quadruplex and two negative peaks at 210 nm and 240 nm (Fig. 1A, middle panel). Consistent with previous studies^32,64,67,68^, the G4 profiles of TERRA and telomeric DNA displayed the strongest G4 features in buffer containing KCl, while buffer containing LiCl resulted in the weakest G4 features (Fig. S1). Several different small molecules, including the acridine derivative BRACO-19 and porphyrin NMM (Fig. 1B) have been shown to interact with DNA and/or RNA G4 structures in vitro^69^ and to regulate telomere functions in vivo^46,50,70^. To determine if these compounds bound with selectivity to TERRA or TeloDNA, we generated fluorescent oligonucleotides for G4 TeloDNA, TERRA, and their antisense oligonucleotides by attaching a 5′ fluorescein label that could be used for fluorescence polarization (FP) assays to measure ligand binding (Fig. 1C,D). We found that BRACO-19 and NMM were able to bind G4 TeloDNA with similar EC_50_ values of 3.9 μM and 4.8 μM (Fig. 1C). The NMM related compound protoporphyrin IX (PP) showed no binding activity for G4 TeloDNA (Fig. 1C). In contrast to G4 TeloDNA, NMM showed increase affinity for TERRA relative to BRACO-19 or PP (Fig. 1D). NMM bound TERRA with EC_50_ of 0.68 μM, while BRACO-19 bound with EC_50_ of 7.4 μM and PP showed no measurable affinity (Fig. 1D). In addition, NMM, but not BRACO-19 or PP altered the CD spectrum of TERRA at its major characteristic peak (~ 260 nm), suggesting it may deform the G4 structure (Fig. 1A, right panel). None of these small molecules showed binding activity for TERRA or TeloDNA antisense oligonucleotides (Fig. S2A and B). We also tested several related small molecules reported to bind G4 DNA, including PM3P, Phen-DC3, acridinium methosulfate (RHPS4), and pyridostatin (PDS) (Fig. S3A–D). Of these, we found that only PDS bound, and with selectivity for TeloDNA (0.05 μM) relative to TERRA (0.66 μM) (Fig. S3E). The selectivity of NMM for TERRA was further corroborated by assaying the relative affinities of these compounds for a non-telomeric RNA G4 structure derived from the Epstein-Barr Virus (EBV) EBNA1 protein mRNA^71,72^. We found EBNA1 G4 RNA bound with PDS (199 nM) and BRACO-19 (227 nM), it did not display any binding with NMM nor with negative control PP (Fig. S4). These findings indicate that only NMM shows significant selectivity for TERRA relative to other G4 RNA or G4 TeloDNA, while other G4 interacting molecules BRACO-19 or PDS, did not show this selectivity.

### TRF2 GAR domain binds selectively to TERRA RNA relative to telomere G4 DNA

**Figure 2:**
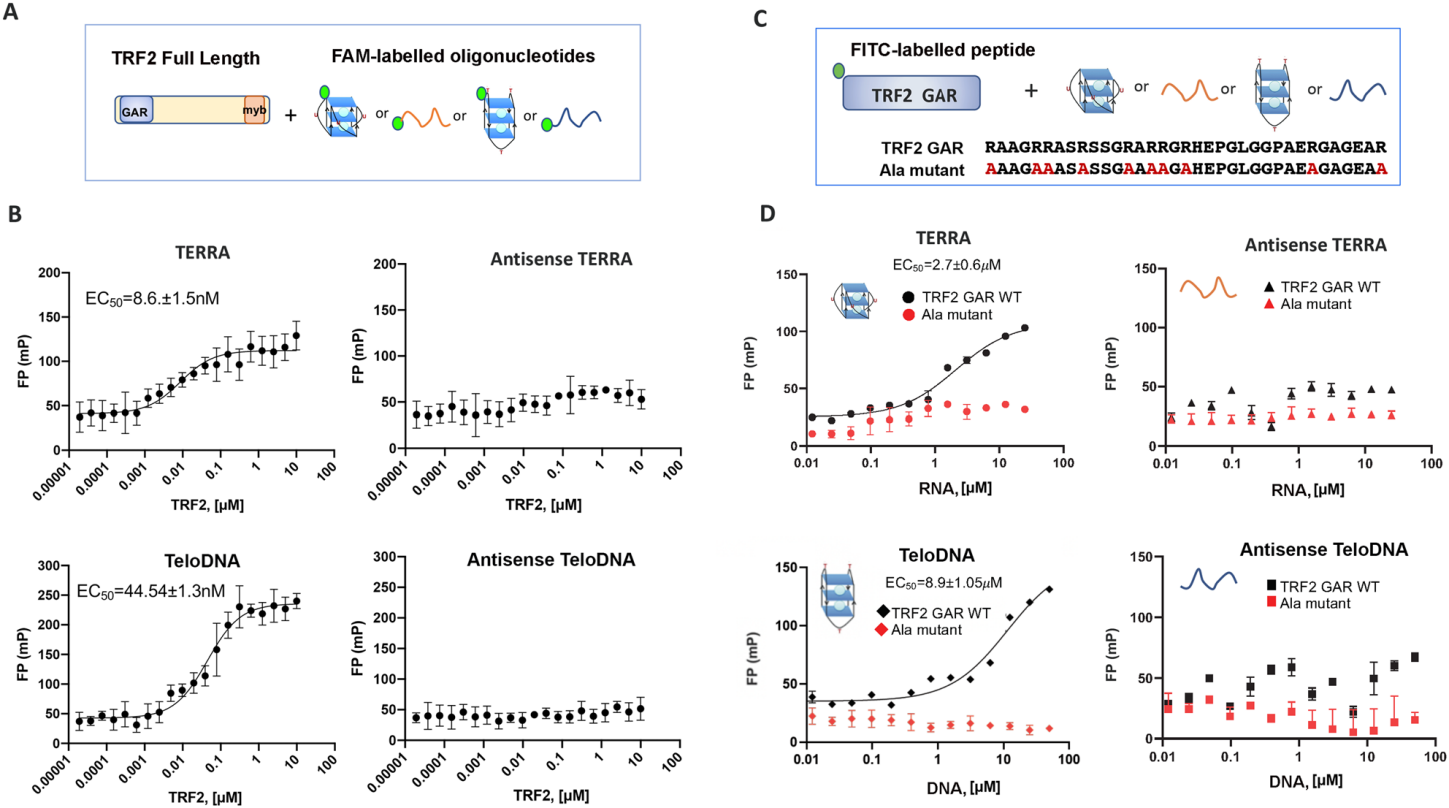
TRF2 GAR binds to G4-containing TERRA and TeloDNA. (**A**) Diagram of FP assay for interaction between FAM labeled nucleic acid oligonucleotides with TRF2 full-length protein. (**B**) FAM labeled TERRA, antisense TERRA, TeloDNA and antisense TeloDNA were incubated with varying amounts of full-length TRF2 protein respectively over the range of concentration shown. All resulting FP values were measured from 3 independent experiments and plotted as a function of oligo concentration using Prism 8.0. EC_50_ were presented in each graph. (**C**) Diagram of FP assay for interaction between FITC labeled TRF2 GAR or Ala mutant peptide with nucleic acid oligonucleotides. (**D**) The TRF2 GAR (black) or Ala mutant (red) peptide (10 nM) was incubated with varying amounts of TERRA G4, antisense TERRA, TeloDNA and antisense TeloDNA, respectively, over the range of concentration shown. All resulting FP values were measured from 3 independent experiments and plotted as a function of oligo concentration using Prism 8.0. EC_50_ were presented in each graph.

The TRF2 GAR domain has been implicated in both TERRA RNA and structure-specific DNA binding^61,73^. We first demonstrated that purified recombinant full-length TRF2 protein can bind preferentially bind with TERRA relative to TeloDNA, and lacks measurable affinity for antisense TERRA or TeloDNA (Fig. 2A,B). To focus on the GAR domain, we synthesized a 35-residue peptide containing the GAR domain of TRF2 and a control peptide with each of the 10 arginine residues mutated to alanine (Ala mutant) (Fig. 2C) and assayed their binding to TERRA RNA or TeloDNA or their antisense nucleic acids by FP assays (Fig. 2D). We found that TRF2 GAR bound TERRA with 2.7 μM affinity (Fig. 2D, upper left), while it bound TeloDNA with 8.9 μM affinity (Fig. 2D, lower left). The Ala mutant showed no detectable binding to either TERRA or TeloDNA. Neither TRF2 GAR nor the Ala mutant showed any detectable binding to the antisense nucleic acids (Fig. 2D, right). The interaction of TRF2 GAR with TERRA and TeloDNA was also measured using Homogenous Time Resolved Fluoresence (HTRF) assay (Fig. S5A and B). These findings are consistent with other reports^64^, and suggest that TRF2 GAR binds selectively to TERRA relative to the other nucleic acids tested.

### NMM preferentially inhibits TRF2 GAR interaction with TERRA relative to telomere G4 DNA

**Figure 3:**
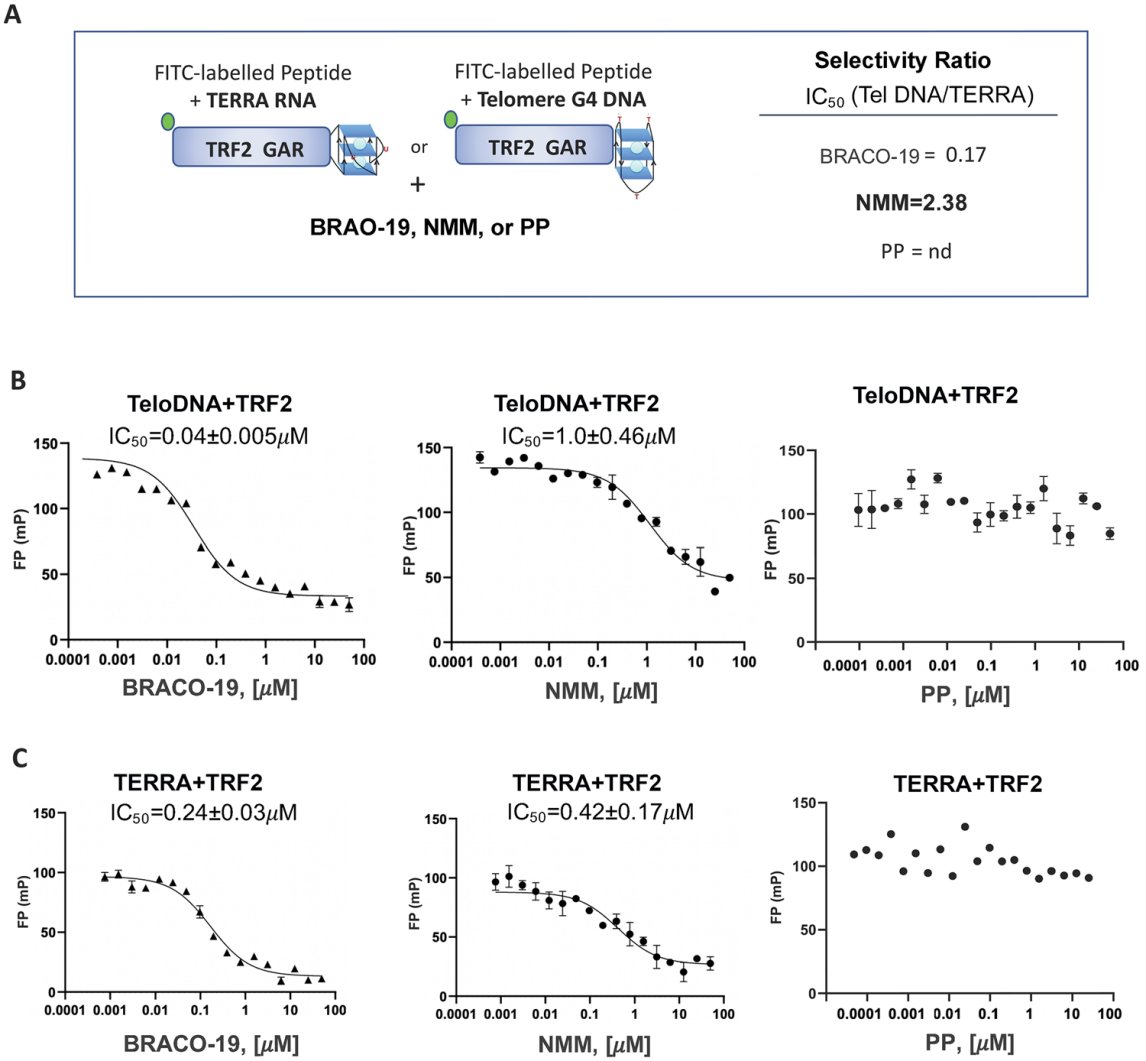
Selective inhibition of TERRA interaction with TRF2-GAR by NMM relative to BRACO-19. (**A**) Diagram of FP assay for disruption of FITC labeled TRF2 GAR interaction with nucleic acid oligos by either BRACO-19, NMM, or PP. Selectivity index for each compound is calculated as the ratio of IC_50_ for inhibiting GAR binding to TelDNA relative to TERRA, using IC50 values from panels (**B**, **C**). (**B**) TeloDNA and TRF2 complex was titrated with BRACO-19, NMM, or PP, respectively over the range of concentrations shown. The IC_50_ are presented in each graph. (**C**) TERRA and TRF2 complex was titrated with BRACO-19, NMM, or PP, respectively, over the range of concentrations shown. The IC_50_ were presented in each graph. All resulting FP values were measured from 3 independent experiments and plotted as a function of compound concentration using Prism 8.0.

We next tested BRACO-19, NMM, or control protoporphyrin PP for their ability to inhibit TRF2 GAR binding to TeloDNA or TERRA (Fig. 3 and S5C and D). Using FP assay, BRACO-19 inhibited TRF2 GAR binding to TeloDNA and TERRA at IC_50_ values of 0.04 μM and 0.24 μM, respectively (Fig. 3B,C). On the other hand, NMM inhibited TRF2 GAR binding to TeloDNA and TERRA at IC_50_ values of 1.0 μM and 0.42 μM, respectively. Calculating a selectivity ratio (SR) of these IC_50_ values suggests that NMM preferentially inhibits TRF2 binding with TERRA (SR = 2.38) relative to BRACO-19 (SR = 0.17). Control compound PP showed no measurable IC_50_ for either TERRA or TeloDNA in these assays (Fig. 3B,C). As a control, none of these small molecules showed an interference on the binding activity of TRF2 GAR with TERRA or TeloDNA antisense oligonucleotides (Fig. S2C and D). In addition, we also observed preferential inhibition of TRF2-TERRA by NMM compared to BRACO-19 using HTRF assay (Fig. S5C and D). Taken together, these findings suggest that NMM preferentially inhibits TRF2 GAR binding to TERRA, while BRACO-19 preferentially inhibits binding to TeloDNA in vitro.

### NMM inhibits TERRA expression

**Figure 4:**
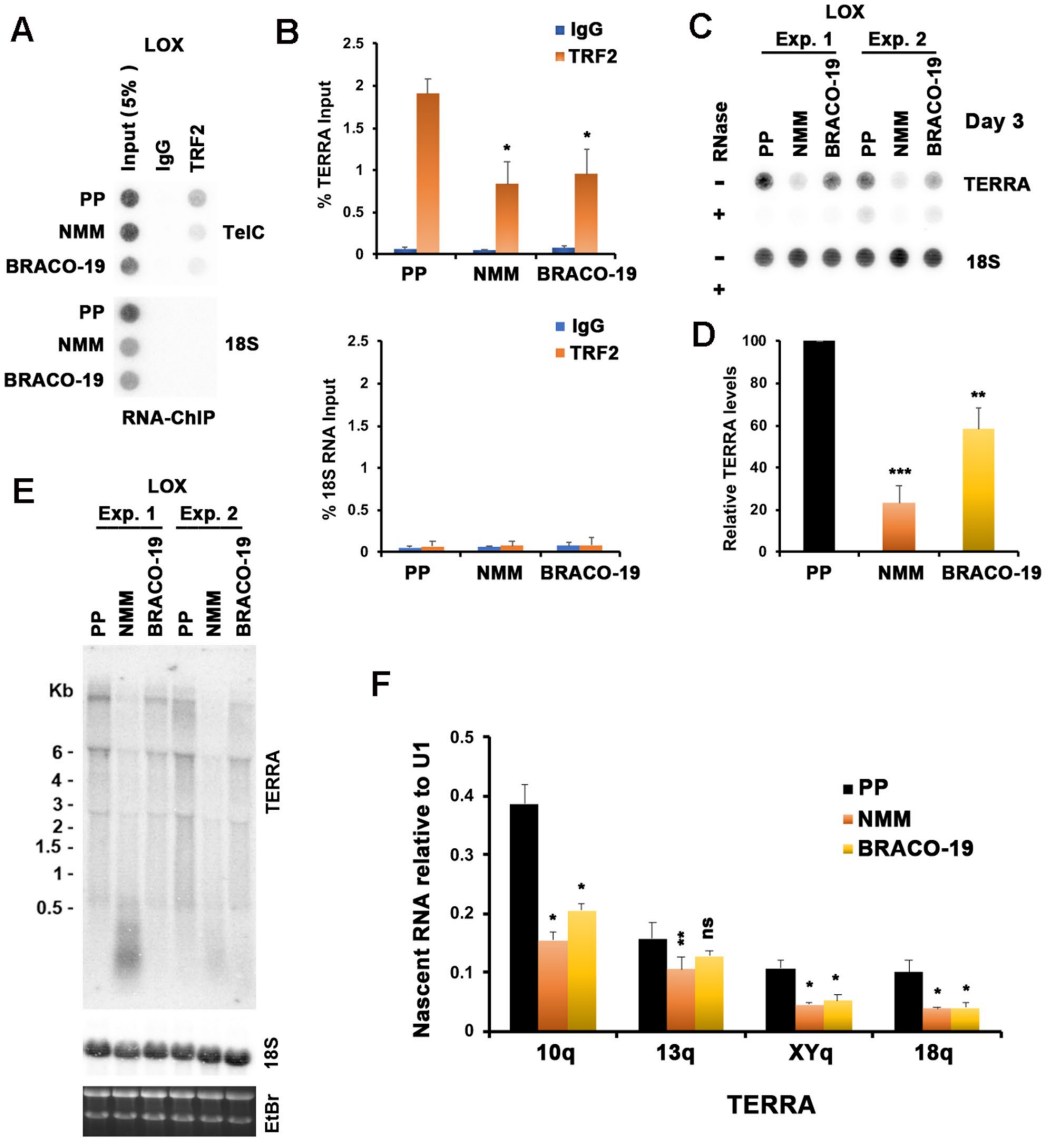
NMM treatment reduces TERRA levels by inhibiting TERRA transcription in vivo. (**A**) RNA ChIP assays were performed on LOX cells treated with 2 μM PP, NMM or BRACO-19 for 24 h. ChIP RNA was detected by RNA dot blot with ^32^P-labeled probes specific for TERRA (top panel) or 18S (bottom panel). (**B**) Quantification of RNA ChIP experiments represented in panel (**A**). ChIP RNA signals were normalized to input RNA signal and shown as a percentage of input. The bar graphs represent the mean and error bars from at least three independent experiments. Error bars indicate SD. Two tailed *t* test, *p value of < 0.05 relative to PP control. (**C**) LOX cells were treated with 2 μM PP, NMM or BRACO-19 for 3 days. Total RNA was isolated and assayed by dot blot using probes containing the (CCCTAA)_4_ (for TERRA), or 18S sequence. 3 μg of RNA was used for each sample and RNase A treatment (+) was used to assess possible DNA contamination. (**D**) Quantification of at least three independent RNA dot blot assays as represented in panel (C). Values are the means and SD (error bars). Two tailed *t* test, **p < 0.005, ***p < 0.001, relative to PP control. (**E**) Total RNA (6 μg) isolated from treated LOX cells from panel (**C**) were analyzed by Northern blot. RNA was detected with probe for TERRA or 18S, as indicated. Ethidium Bromide (EtBr) staining of Northern gel was shown in bottom panel to indicate the stability of total RNA. (**F**) LOX cells were treated with 2 μM PP, NMM or BRACO-19 for 2 days, and then pulse labeled with 0.2 mM EU for 4 h followed by RNA isolation, Click-iT biotinylation and streptavidin purification of nascent RNA. Nascent RNA was then quantified by RT-qPCR with primers specific for chromosome-specific TERRA from 10q, 13q, XYq, and 18q and normalized relative to U1 RNA. Values are the means and SD (error bars). Two tailed *t* test, **p < 0.05, ***p < 0.01, ns, not significant relative to PP control.

To determine if NMM or BRACO-19 affect TRF2 binding to TERRA in living cells, we compared the ability of these compounds, along with the PP control, to alter the TRF2-TERRA interaction by RNA-ChIP assay (Fig. 4A,B). For these experiments, we used LOX human melanoma cells that are telomerase positive with long telomeres and high levels of TERRA^74^. For RNA-ChIP, we treated cells with 2 μM of each compound for 24 h. Both NMM and BRACO-19 inhibited TRF2 interaction with TERRA by ~ 50% relative to PP control (Fig. 4B). Neither NMM, PP, nor BRACO-19 had any significant effect on TRF2 binding to telomere DNA by ChIP-assay (Fig. S6A and B). Remarkably, NMM and to a lesser extent BRACO-19 treatment reduced total TERRA levels relative to PP control as measured by RNA Dot blot (Fig. 4C,D) and Northern blot (Fig. 4E). Furthermore, NMM and BRACO-19 reduced TERRA levels in LOX and ALT positive U2OS cells (Fig. S6C and D), indicating that the reduction in TERRA levels is not specific to cell type or telomerase expression status. We also found that PDS, which bound TERRA and TeloDNA G4 similar to BRACO-19, also reduced TERRA levels in LOX treated cells (Fig. S6E and F). Northern blot indicated that TERRA levels were not only reduced by NMM, but accumulated as a shorter form, which was not observed with BRACO-19 or PP treatment (Fig. 4E). To determine if the nascent transcription of TERRA was affected, we treated LOX cells with either PP, NMM, or BRACO-19 for 48 h prior to pulse labeling with ethynyl uridine (EU) for 4 h after prior treatment with either PP, NMM, or BRACO-19 (Fig. 4F). EU labeled RNA was recovered by Click-iT biotin capture and then assayed by RT-qPCR for chromosome specific TERRA RNA. We found that both NMM and BRACO-19, but not PP led to a significant reduction (~ two fold) in nascent TERRA transcripts from chromosomes 10q, XYq and 18q (Fig. 4F). These findings suggest that G4 interacting molecules are inhibiting the nascent transcription of TERRA.

### NMM induced telomere DNA shortening and fragility

**Figure 5:**
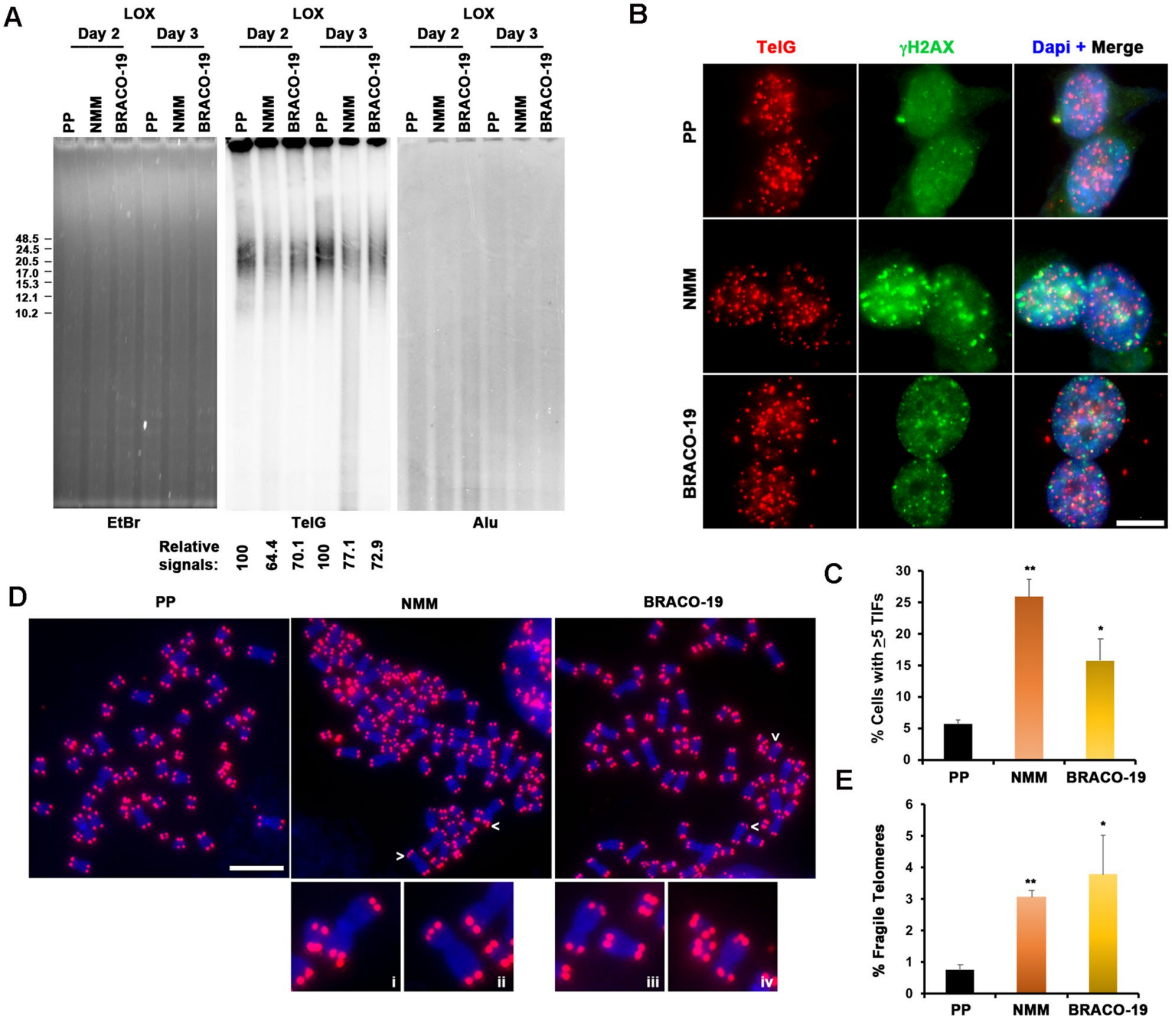
NMM and BRACO-19 lead to telomere signal loss and induce telomere dysfunction and fragility. (**A**) Telomere length analysis of LOX cells treated with 2 μM PP, NMM or BRACO-19 for 2 and 3 days. Telomere length and relative amount of telomeric DNA was determined by restriction digestion of genomic DNA with AluI/MboI, followed by PFGE and Southern hybridization with a ^32^P-labeled (TTAGGG)_4_ probe (middle panel) followed by a ^32^P-labeled Alu probe (right panel). Ethidium bromide staining of total DNA digest was used to indicate for equal DNA loading (left panel). Fragment size (in kb) is indicated on left of blot. Relative intensity of telomeric DNA signals relative to PP treated cells is indicated below blot. (**B**) LOX cells were treated with 2 μM PP, NMM or BRACO-19 for 3 days, as indicated, and assayed by IF FISH for TIF formation. γH2AX foci was shown in green and telomere DNA foci are shown in red. The merged and DAPI counterstained images are shown in the right panels. Scale bar, 10 mm. (**C**) Quantification of TIFs by chemical treatments as shown in panel (**B**). Cells with five or more γH2AX foci colocalizing with Tel DNA were scored as TIF positive. The bar graph represents the mean and SDs from three independent TIF assays (> 100 cells were scored). *p value of < 0.05, **p value of 0.01. (**D**) Representative telomere FISH analysis on metaphase spreads of LOX cells grown for 3 days in the presence of 2 μM PP, NMM or BRACO-19. Telomere signals were shown in red and DAPI staining of metaphase chromosomes were shown in blue. White arrows indicate fragile telomeres. Magnified views of fragile telomeres found in NMM (I, ii) or BRACO-19 (iii, iv) treated cells were shown in below. Scale bar, 10 μm. (**E**) Quantification of fragile telomeres in LOX cells treated with 2 μM PP, NMM or BRACO-19 for 3 days. Data shown were obtained from three independent experiments. Student’s *t* test was used for statistical analysis. Error bars indicate SD. *p value of < 0.05, **p value of < 0.01.

We next assayed the effect of these G4 interacting molecules on telomere repeat DNA. Telomere repeat length and signal intensity were first measured by telomere restriction length assay (TLA). This revealed that NMM, and to a lesser extent BRACO-19 and PDS, reduced telomere repeat DNA signals (Fig. 5A and S6G). NMM also led to a decrease in average telomere length, which was more significant after 3 days of treatment (Fig. 5A). To determine if G4 telomere DNA was affected by incubation with G4 interacting drugs, we performed ChIP assay with BG4 antibody that is known to be highly specific for G4 DNA, but not RNA^75,76^. We found that LOX cells treated with NMM had significant reduction in G4 telomere DNA relative to PP or PDS treated cells (Fig. S6H). These findings are consistent with the loss of telomere signal measured in TLA assay.

We next assayed the effects of PP, NMM, and BRACO-19 on DNA damage signaling by γH2AX or 53BP1 immunofluorescence (IF) combined with telomere FISH. We noted that NMM induced a high level of γH2AX or 53BP1 associated telomere dysfunction-induced foci (TIFs), while BRACO-19 produced a moderate level, and PP was equivalent to background for LOX cells (Fig. 5B,C, and Fig. S7A and B). Scoring for γH2AX colocalization with telomere DNA signal revealed that NMM induced a ~ fivefold increase in telomere-associated γH2AX compared to PP treatment, whereas BRACO-19 induced a ~ three fold increase relative to PP treatment (Fig. 5B,C). We also assayed for telomere aberrations by metaphase chromosome FISH (Fig. 5D,E). We observed that both NMM and BRACO-19 treatment increased the frequency of telomere aberrations, including the appearance of fragile telomere doublets (Fig. 5D,E), which are typically associated with defects in telomere DNA replication^61,77,78^.

### TRF2 GAR domain is required for TERRA expression

**Figure 6:**
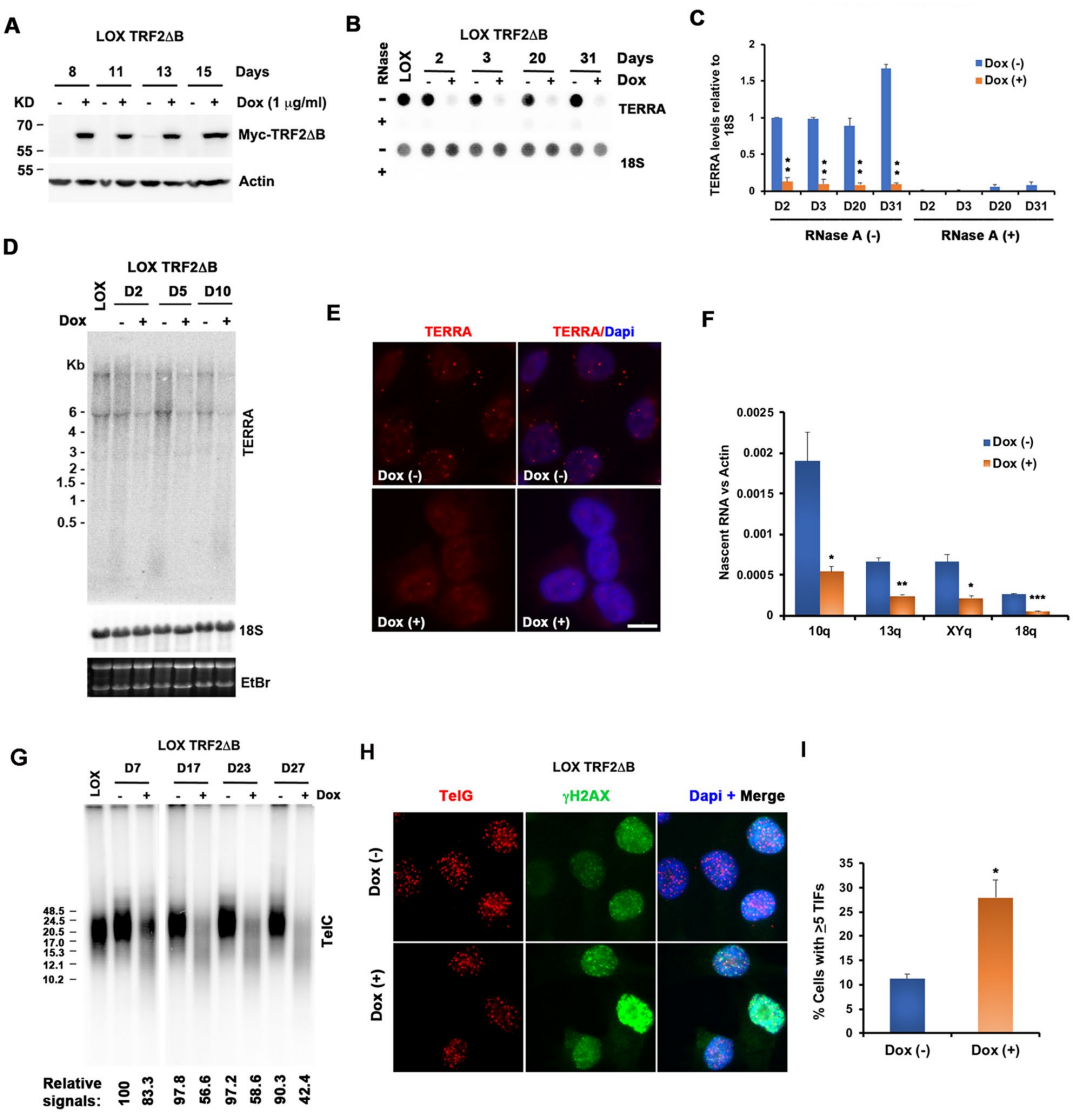
TRF2 GAR domain is required for TERRA expression, telomere length stability, and DNA damage protection. (**A**) Western blot of cell lysates of LOX cell line stably expressing Dox inducible TRF2ΔB. Myc-tagged TRF2ΔB and Actin in the absence (−) or presence (+) of Dox (1 μg/ml) for indicated days are shown. (**B**) RNA dot blot was used to measure TERRA levels in LOX TRF2ΔB cells during the course of Dox induction. Total RNA was isolated and assayed using ^32^P-labeled probes specific for TERRA or 18S RNA. 3 μg of RNA was used for each sample and RNase A treatment (+) was used to assess possible DNA contamination. (**C**) Quantification of at least three independent RNA dot blot assays as represented in panel (**B**). Values are the means and SD (error bars). **p value of < 0.01. (**D**) Total RNA (6 μg) isolated from LOX cells and LOX TRF2ΔB cells was analyzed by Northern blot. RNA was detected with probe for TERRA, or 18S, as indicated. Ethidium Bromide (EtBr) staining of Northern gel is shown in bottom panel to indicate the stability of total RNA. (**E**) RNA FISH analysis of LOX TRF2ΔB cells in the absence (−) or presence (+) of doxycycline (1 μg/ml) for 3 days. TERRA RNA foci were assayed with a PNA-Cy3-(CCCTAA)_3_ probe and shown in red. The merged and DAPI counterstained images are shown in the right panels. Scale bar, 10 mm. (**F**) LOX TRF2ΔB cells treated with (+) or without (−) doxycycline for 2 days, and then pulse labeled with 0.2 mM EU for 4hrs followed by total RNA isolation, Click-iT biotinylation and streptavidin purification of nascent RNA. Nascent RNA was then quantified by RT-qPCR with primers specific for chromosome-specific TERRA from 10q, 13q, XYq, and 18q, and normalized relative to Actin RNA. Values are the means and SD (error bars). Two tailed *t* test, **p < 0.05, ***p < 0.01, ***p < 0.001, relative to Dox (−) control. (**G**) Telomere length analysis of LOX TRF2ΔB cells during the course of Dox induction. Telomere length and relative amount of telomeric DNA was determined by restriction digestion of genomic DNA with AluI/MboI, followed by PFGE and Southern hybridization with a ^32^P-labeled (CCCTAA)_4_ probe. Fragment size (in kb) is indicated on left of blot. Quantification of total telomere repeat signals for each sample as shown below each lane. Telomere signal represents the telomeric signals relative to Dox (−) at day 7, which was arbitrarily set at 100. (**H**) LOX TRF2ΔB cells were cultured in the absence (−) or presence (+) of doxycycline (1 mg/ml) for 5 days and assayed by IF FISH for TIF formation. γH2AX foci are shown in green and telomere DNA foci was shown in red. The merged and DAPI counterstained are shown in the right panels. Scale bar, 10 μm. (**I**) Quantification of TIFs after TRF2ΔB expression as shown in panel (**H**). Cells with five or more γH2AX foci colocalizing with Tel DNA were scored as TIF positive. The bar graph depicts the mean and SDs from three independent TIF assays (> 100 cells were scored). *p value of < 0.05.

To better understand the function of the TRF2 GAR domain and its potential role in mediating the telomeric effects of these G4 interacting drugs, we generated a stable LOX cell line with a doxycycline (Dox)-inducible TRF2ΔB gene. TRF2ΔB expression was readily detectable within 3 h after Dox-induction (Fig. S8C) and continuously expressed in cells treated for more than 2 weeks (Fig. 6A). The effect of TRF2ΔB on TERRA levels was analyzed by Dot blot (Fig. 6B,C), Northern blot (Fig. 6D) and RNA-FISH (Fig. 6E). All assays clearly indicated that TRF2ΔB caused an abrupt loss of TERRA expression, similar to that observed with NMM. Dot blot showed ~ tenfold decrease in total TERRA levels by 2 days after Dox-induction of TRF2ΔB and the decreased TERRA levels were not recovered during the time course (Fig. 6B,C). Northern blot revealed a similar decrease in total abundance with the accumulation of smaller forms of TERRA by 10 days after Dox-induction (Fig. 6D). RNA-FISH at 3 days after Dox-induction confirmed the decrease in TERRA in intact cells (Fig. 6E). To rule out the possibility of an effect of doxycycline on TERRA levels, we assayed LOX and LOX TRF2ΔB cells cultured in the absence or presence of Dox for 2 days. RNA dot blot showed that Dox treatment had no effect on TERRA in LOX cells, indicating that the loss of TERRA depends on TRF2ΔB induction (Fig. S8A). In addition, the reduction of TERRA levels was detectable at 3 h after Dox-induction and showed progressively reduced levels across the 24 h time frame (Fig. S8B). In agreement with the previous finding that TRF2ΔB expression induces growth arrest phenotypes^79^, Western blot indicated the gradual increase of p53 and p21 protein along the course of TRF2ΔB induction, while control total histone H3 or H3K9me3 showed no obvious change (Fig. S8C). To determine if the effect of TRF2ΔB on TERRA levels was not cell type-specific, we assayed TERRA levels by Dot blot (Fig. S8D) and Northern blot (Fig. S8F) in U2OS cells containing the Dox-inducible TRF2ΔB gene. Western blot showed clear TRF2ΔB expression after Dox treatment in U2OS TRF2ΔB cells (Fig. S8E). Consistent with our findings in LOX TRF2ΔB cells, both RNA dot blot and Northern blot revealed that TRF2ΔB induction resulted in a time-dependent decrease of TERRA signals in U2OS cells. To determine if this decrease in TERRA was due to changes in nascent transcription, we induced TRF2ΔB prior to pulse labeling with EU followed by Click-iT affinity purification and RT-qPCR for analysis of chromosome specific telomere transcripts (Fig. 6F). Similar to results with NMM treatment, we found that TRF2ΔB expression led to a loss of nascent TERRA RNA from all chromosomes tested (Fig. 6F). Taken together, these data indicate that TRF2ΔB induces a loss of TERRA nascent transcription that is not dependent on cell type or telomere maintenance mechanism.

### TRF2ΔB induces telomere DNA loss and DNA-damage foci

TRF2ΔB expression also led to a substantial loss of telomere repeat DNA signal and decrease in average length, as measured by pulse field gel analysis of Southern blot (Fig. 6G). Telomere signal loss and average length reduction was apparent by day 7 after Dox induction and decreased further through day 27. In addition, LOX cells expressing TRF2ΔB showed increase in γH2AX signals colocalized with telomere foci (Fig. 6H,I). Quantification of these foci indicated ~ 2.5-fold increase in cells with ≥ 5 TIFs by 5 days after Dox-induction. We also assayed for telomere aberrations by metaphase chromosome FISH in LOX TRF2ΔB cells treated with Dox for 20 days (Fig. S8G). We found that TRF2ΔB induction increased the frequency of telomere aberrations, including the appearance of fragile telomeres and loss of telomere signals^80^. Taken together, these findings indicate that TRF2ΔB phenocopies many of the telomere and TERRA defects observed with NMM treatment.

## Discussion

G4 DNA and RNA have been implicated in the regulation of diverse cellular processes, as well as potential for pathogenic roles in human disease^27,81–84^. TERRA can form G4 structures, but the biological role of these G4 structures are not well-understood. Here, we show that the G4 structure of TERRA is recognized by the TRF2 basic GAR domain, and that this interaction is important for TERRA stability and telomere DNA maintenance^61^. We also found that the G4-interacting compound NMM has selectivity towards TERRA RNA and inhibits the interaction between TERRA and TRF2 GAR. Furthermore, we demonstrate that NMM phenocopies genetic deletion of the TRF2 basic domain in regulating TERRA expression and telomere DNA instability.

G4 DNA and RNA can form a range of structures that have different functions and affinities for protein and small molecule ligands^85^. Our FP assays showed that NMM preferentially bound G4 TERRA relative to G4 TeloDNA (Fig. 1). Both NMM and BRACO-19 showed similar affinities for G4 TeloDNA, but only NMM showed preferential binding to TERRA (Fig. 1). NMM is reported to have high selectivity to G4 DNA over other DNA structures and bind selectively to parallel forms of G4 DNA^54,57,86^. TERRA is reported to form a parallel stranded G4^32,87^, and also to have a high affinity for related porphyrins, such as TMPyP4^88^. In contrast, BRACO-19 and PDS bound G4 DNA preferentially over TERRA, indicating that both G4 RNA and DNA have different affinities for these small molecules. Our FP data also demonstrated that TRF2 GAR peptide binds preferentially to G4 TERRA relative to G4 TeloDNA (Fig. 2). It was reported that the GAR motif facilitates the folding of G4 telomeric DNA^89,90^. The G4 structure of TERRA was shown important for its binding to TRF2^64^. Therefore, the recognition of the TERRA G4 structure by TRF2 GAR is likely to be an important functional interaction in telomere organization and regulation.

G4 interacting molecules are known to induce telomere-specific defects in vivo^46,91,92^. Others have reported that BRACO-19, PDS, and NMM can inhibit telomerase activity through stabilization of telomeric G4 DNA^49,50,70,93^. We found NMM, PDS, and BRACO-19 cause the rapid loss of telomere repeat DNA (Fig. 4 and S6) and a corresponding increase in telomere DNA damage signal marked by colocalization with γH2AX and fragile telomere doublets (Fig. 5). While both NMM and BRACO-19 had similar activity, we observed more potent inhibition of TRF2 interaction with TERRA by RNA-ChIP (Fig. 4A,B) and a more robust loss of TERRA (Fig. 4C–E) with NMM than BRACO-19. NMM also showed a more rapid and extensive loss of telomere repeat DNA (Fig. 5A). While inhibition of telomerase may also contribute to loss of telomere signal, the rapid nature of these effects indicate they are most likely due to perturbation of telomere structure and DNA replication defects. We suggest that the specific effect of NMM on TERRA levels is most consistent with NMM’s selective binding to TERRA and disruption of its interaction with the TRF2 GAR domain.

To better understand the effects of NMM on the TRF2 GAR domain interaction with TERRA, we compared NMM effects with those of ectopic expression of TRF2ΔB (Fig. 6), We found that TRF2ΔB expression phenocopied many of the observed effects of NMM, including the rapid loss of TERRA, loss of telomere DNA length and signal, and increase in telomere DNA damage signaling, including fragile telomeres. The rapid loss of telomeric DNA and the appearance of fragile telomeres supports the model that the TRF2 GAR domain is important for the completion of telomere DNA replication. We have previously reported that TRF2 GAR is required for TERRA binding to recruit ORCs to telomeres and facilitate telomere DNA replication and stability^61^ and more recently shown a direct role of TRF2 GAR in initiation of DNA replication within telomere repeat DNA^80^. Both TRF2 GAR and TERRA have been implicated in telomere DNA replication, as well as DNA conformation and heterochromatin formation^27,94–96^. Our new findings suggest that disruption of TRF2 GAR interaction with TERRA leads to a rapid loss of TERRA, followed by the subsequent disruption of telomere replication and consequent telomere repeat loss and DNA damage signaling.

TRF2 has multiple domains with distinct activities. Other studies have shown ectopic expression TRF2 dominant negative homodimerization domain, as well as depletion of TRF2, reduced TERRA expression^18^, consistent with our findings that TRF2 is required for TERRA expression. The TRF2 GAR domain has been shown to bind the 4-way junctions formed at T-loops and restrict promiscuous telomere recombination^96^. TRF2 was also shown to inhibit telomerase expression in normal cells via its interaction with G4 structures in the TERT promoter, although it is not clear if the GAR domain was required for this activity^97^. TRF2 may interact with G4 DNA through domains other than GAR, and the GAR domain is known to have functions in addition to TERRA binding. Another study found that another G4 interacting molecule CK1-14 bound TERRA and disrupted TRF2 binding to telomere repeat DNA^65^. In contrast, we found that NMM did not disrupt TRF2 binding to telomeric DNA, but did inhibit TRF2 binding to TERRA. We also found that NMM and BRACO-19 inhibit the nascent transcription of TERRA. How these multiple interactions and functions are coordinated to regulate telomere structure and replication will require further investigation.

In conclusion, we find that G4 interacting molecules can have selectivity for different G4 structures, including selectivity for TERRA relative to telomere G4 DNA. These and newer generation G4 interacting molecules may be useful as probes to better dissect functions of telomere G4 structures in vivo. A recent study with a different class of G4 interacting molecule was found to bind selectively to TERRA and allosterically inhibit TRF2 binding to telomere DNA to promote apoptosis in cancer cells^65^. Furthermore, small molecule inhibition of TERRA is being explored for cancer treatment^21,27,98^. Our findings suggest that the TERRA interaction with TRF2 GAR is responsive to small molecule inhibition, and may have potential utility for telomere-based therapies.

## Materials and methods

### Oligos and chemicals

Nucleic acids of TERRA (UUAGGG)_4_, TERRA control (CACUGA)_4_, TERRA antisense (CCCUAA)_4_, G4 DNA (TTAGGG)_4_, DNA antisense (CCCTAA)_4_ were purchased from Integrated DNA Technology (IDT) with desalting purification. TRF2 peptide (RAAGRRASRSSGRARRGRHEPGLGGPAERGAGEAR), Ala mutant (AAAGAAASASSGAAAAGAHEPGLGGPAEAGAGEAA), with or without 5′ Fluorescein isothiocyanate (FITC) was purchased from EZbiolab with > 95% purity. G-quadruplex interacting small molecules: *N*-methyl mesoporphyrin IX (NMM), protoporphyrin IX (PP) were purchased from Frontier Scientific, and BRACO-19 was synthesized by Cynthia Meyers (Fox Chase Cancer Center).

### Plasmid construction

The doxycycline-inducible lentiviral plasmid used to express N-terminally Myc-tagged TRF2∆B, a TRF2 mutant lacking the B domain (aa 1–44), was generated as follows. The inducible expression lentiviral vector pInducer10^99^, (a gift from W Guo, Albert Einstein College of Medicine, NY) was digested with AgeI and MluI, and a linker containing XhoI and KpnI-compatible BstXI sites inserted between the AgeI-MluI sites to generate pInducer10L^100^. The human TRF2∆B cDNA sequence was excised from pLPC-NMYC TRF2∆B (gift from Titia de Lange, Addgene plasmid # 16,067) with XhoI and KpnI and inserted into pInducer10L, directly downstream of the doxycycline-inducible promoter, to generate pIND-MYC-TRF2∆B.

### Cell cultures

LOX human melanoma cells^74,101^ were grown in complete RPMI (high glucose RPMI supplemented with 10% heat inactivated FBS, 100 I.U./mL Penicillin, and 100 μg/mL Streptomycin (Corning)). LOX cells inducibly expressing TRF2∆B protein were generated by lentiviral transduction. Cells were infected with lentiviral particles containing pInducer10L and pIND-MYC-TRF2∆B, and single cell clonal colonies were selected in 1 µg/mL puromycin. N-terminally Myc-tagged TRF2∆B protein was expressed in stable lines by induction with 1 µg/ml doxycycline at indicated days. Due to the instability of doxycycline in the culture, the culture medium will be replaced with fresh doxycycline every 2 days. U2OS cells were grown in complete DMEM supplemented with 10% FBS, 100 I.U./mL Penicillin, and 100 μg/mL Streptomycin (Corning). U2OS cells inducibly expressing TRF2∆B protein were generated by lentiviral transduction, as described above in LOX cells. All cells were cultured in a 5% CO_2_ incubator at 37 °C. For chemical treatment, cells were cultured in the medium containing 2 μM PP, NMM, BRACO-19 or PDS for 48 h or 72 h.

### Circular dichroism (CD) spectroscopy

All nucleic acids were dissolved in buffer containing 20 mM HEPES pH7.5, 150 mM salt (KCl or LiCl), 20% PEG8000 at the concentration of 10 μM for experiments. A J-815 spectrometer equipped with a PFD-425S Peltier cell holder was used to measure CD spectra at 4 °C between 200 and 340 nm to determine the secondary structure content of various oligo constructs. The molar ellipticity was calculated and the plot was made using Excel.

### Fluorescence polarization (FP) assay

All the nucleic acids, peptides and chemicals were dissolved in buffer containing 20 mM HEPES pH7.5, 150 mM salt (KCl, NaCl, LiCl) at the stock concentration of 1 mM. For peptide and nucleic acid interactions, 25μL of each nucleic acids with a sequential twofold dilutions starting from 50 μM was added into each well of 96-well plate followed by adding 25μL of 10 nM FITC labeled TRF2 peptide. For compounds and nucleic acid interactions, 20μL of each compounds with a sequential twofold dilutions starting from 50 μM were added into each well of 96-well plate followed by adding 5μL of 20 ng/mL tRNA and 25μL of 10 nM FAM labeled nucleic acids. For inhibition assay, 5μL FITC labeled TRF2 GAR peptide (10 nM final concentration) and 10 μL oligos (5 μM final concentration) were mixed, followed by adding 5μL of each compound with a sequential twofold dilutions starting from 50 μM into each well of 384-well plate. All components were mixed thoroughly, and the plate was incubated at room temperature for 1 h before placing in an Envision Plate Reader for reading at 495/520 nm. All data curves were fitted using Prism 8.0.

### Western blotting

Cells were lysed in RIPA buffer (150 mM NaCl, 50 mM Tris, 0.5% Na-Deoxycholate, 0.1% SDS and 1% NP-40), and equal amount of protein was resolved using 8–16% Tris–Glycine gel (ThermoFisher Scientific). Primary antibodies used were Actin (Sigma), p53 (Sigma), p21 (Abcam), histone H3K9me3 (Diagenode), histone H3 (Millipore), and Myc (Cell Signaling). Secondary antibodies used were goat anti-mouse HRP (1:1,000, Sigma). Antibody signal was detected using Luminata HRP detection reagent (Millipore) and Luminescent Imager 680 (Amersham Biosciences).

### Telomere and TERRA analysis

TERRA dot blot and Northern blot analyses were described previously^61^. Telomere length analysis, telomere IF and metaphase FISH were described previously^61,102^. Additional details are provided in Supplemental Information.

### Statistics

Statistical analyses were carried out by paired two-tail Student’s *t* tests. *p* values and significance levels are annotated in the figures and described in the figure legends.

## Supplementary Information

Supplementary Information.
