# Supplementary material for: TERRA G-quadruplex RNA interaction with TRF2 GAR domain is required for telomere integrity
Source: Sci Rep. 2021 Feb 10;11:3509. doi: 10.1038/s41598-021-82406-x (PMC7876106; doi:10.1038/s41598-021-82406-x)

SUPPLEMENTARY INFORMATION

TERRA G-Quadruplex RNA Interaction with TRF2 GAR Domain Is Required for Telomere Integrity

Yang Mei [1*], Zhong Deng [1*], Olga Vladimirova [1], Nitish Gulve[1], F. Brad Johnson[2], William C. Drosopoulos [3], Carl L. Schildkraut [3], and Paul M. Lieberman[1,4]

[1]The Wistar Institute
Philadelphia, PA 19104
[2]Department of Pathology, Perelman School of Medicine at the University of Pennsylvania, Philadelphia, PA 19104
[3]Department of Cell Biology, Albert Einstein College of Medicine
1300 Morris Park Avenue, NY 10461

[4] Corresponding Author
Lieberman@wistar.org
215-898-9491

Supplementary Methods

Homogenous Time Resolved Fluorescence (HTRF) assay

All material including peptide, nucleic acids and compounds were dissolved in buffer containing 20mM HEPES pH7.5 150mM KCl. To measure the direct interaction between peptide and nucleic acids, 5μL of FITC-labeled TRF2 GAR peptide was added into each 384 well with a sequential of 2-fold dilution starting from 200nM, 5μL of 10nM biotin-labeled nucleic acid was then added into the well and fixed with the peptide, 5μL of buffer was added to make each well contain 15μL solution. The plate was incubated at room temperature for 30 minutes, following by adding 5μL of streptavidin-Tb-cryptate (Cisbio, PerkinElmer) and the plate was then incubated at room temperature for another 30 minutes before measured by EnVision plate reader (330/520 nm). To measure the compound disruption on peptide and nucleic acids, 5μL of small molecule was added into each 384 well first with a sequential of 2-fold dilution starting from 5μM, followed by 5μL of 10nM nucleic acid and 100nM peptide and then the plate was incubated at room temperature for 30 minutes. The rest step was similar to the direct binding measurement assay. The curves were analyzed using Prism 8.0.

RNA dot-blot and Northern blot

Total RNA was isolated as previously described by using TRIzol reagent (Invitrogen) [1]. RNA samples were treated with 1 U DNase I for 30 min at 37 °C, followed by DNase I inactivation in the presence of EDTA at 65 °C for 5 min. RNA concentration was determined by Nanodrop-2000 (ThermoFisher). RNA dot-blot was performed as previously described [1]. Briefly, 3 μg RNA was denatured, blotted to GeneScreen Plus Nylone membrane (Perkin-Elmer), and UV crosslinked at 125 mJ in UV Stratalinker 2400 (Stratagene). For RNase A treatment, 3 μg RNA was incubated with RNase A at a concentration of 100 μg/ml at 37 °C for 1h, followed by dot-

blot procedures. Northern blot was performed as previously described with some modifications [2]. Briefly, 6 μg RNA was denatured in NorthernMax formaldehyde loading dye (Ambion) for 15 min at 65 °C and separated by 1.2% agarose formamide gel in 1X Mops buffer (Ambion) at 5V/cm for 2-3 hrs. RNA samples were transferred to GeneScreen Plus blotting membranes (Perkin-Elmer) with 10X SSC and UV cross-linked onto membranes at 125 mJ in UV Stratalinker 2400 (Stratagene). The hybridization was performed using a ^{32}P-labeled TERRA probe in Church buffer (0.5 N Na-phosphate, pH 7.2, 7% SDS, 1 mM EDTA, 1% BSA) for overnight at 50 °C. The blot was washed twice in 0.2 N Na-phosphate, 2% SDS, and 1 mM EDTA at room temperature and once in 0.1 N Na-phosphate, 2% SDS, and 1 mM EDTA at 50 °C. Images were collected by phosphor- imager (Amersham Biosciences), and were visualized with a Typhoon 9410 Imager (GE Healthcare) and quantified with ImageQuant TL software (GE Healthcare). To reprobe the blot, the membrane was stripped in 1% SDS, 0.1× SSC, and 40 mM Tris·HCl (pH 7.5) at 80 °C and hybridized with a ^{32}P-labeled 18S probe under the same conditions. The probes were 5′ end-labeled with γ-^{32}P-ATP by T4 polynucleotide kinase (NEB) per the manufacturer's instructions. The probe sequences are 5′-TAACCCTAACCCTAACCCTAACCC-3′ for TERRA, and 5′-CCATCCAATCGGTAGTAGCG-3′ for 18S.

RNA and DNA ChIP

RNA ChIP assays were performed as described [1] with minor modifications. Briefly, 1 x 10^7 LOX cells treated with indicated chemicals for 24 hr were cross-linked for 10 mins with 1% formaldehyde, and cross-linking was quenched by adding glycine to a final concentration of 125mM. The cell pellet was washed with cold PBS, resuspended in 500 μl of Buffer A (5mM PIPES, pH 8.0, 85mM KCl, 0.5% *NP40,* protease inhibitors cocktail, 50U/ml SUPERase•in), and placed on ice for 10 minutes. The nuclei were collected by centrifugation at 5000 rpm for 5 mins

at 4 °C, washed in Buffer A without NP-40, and resuspended in 500 µl of Buffer B (1% SDS, 10mM EDTA, 50mM Tris-HCl pH 8.1, protease inhibitors cocktail, 50U/ml SUPERase.In) on ice for 10 mins. The lysates were sonicated, cleared by centrifugation, and supernatants were diluted 10 fold into IP Buffer (0.01% SDS, 1.1% Triton X-100, 1.2mM EDTA, 16.7mM Tris pH 8.1, 167mM NaCl, protease inhibitors cocktail, 50U/ml SUPERase•in) for IP reaction with rabbit IgG (Santa Cruz Biotechnology) or rabbit polyclonal anti-TRF2 (Pocono Rabbit Farm) at 4 °C overnight. Each immune complex was collected by Dynal A beads (Invitrogen), washed five times (1 ml wash, 10 mins each) in ChIP related wash buffer at 4 °C, eluted by addition of 200µl Elution buffer (10mM Tris, pH 8.0, 5mM EDTA, 1% SDS, 50U/ml SUPERase.In) at 70 ºC for 20 mins, and the elutes were placed at 70 ºC for 2 hrs to reverse cross-linking. RNA was isolated with Trizol reagent and resuspended in 50µl of DEPC-treated water followed by DNaseI treatment and RNA dot-blot. DNA ChIP assays was performed as described previously [3] with minor modifications. Briefly, 1 x 10^7 LOX cells treated with indicated chemicals (2 µM) for 48 hrs were subject to ChIP analysis and immunoprecipitated DNA was assayed by DNA dot-blot using ^{32}P-labeled $(TTAGGG)_4$ or Alu probe.

Telomere length analysis

Genomic DNA was isolated from cells using a genomic DNA purification kit (Promega) and digested with AluI and MboI. Digested genomic DNA was purified by phenol/choloroform extraction and quantified using a Qubit 4 Fluorometer (Thermal Scientific). Equal amounts of digested DNA (5 µg) were separated by pulse-field gel electrophoresis (PFGE) performed with a 1% agarose gel at 6V/cm, switch times from 1 to 12s, 15˚C for 12h using CHEF-DRIII system (Bio-Rad). The DNA was transferred to GeneScreen Plus blotting membranes (Perkin-Elmer) and analyzed by Southern blot. The blots were hybridized with a ^{32}P-labeled telomere repeat or

Alu probe at 42˚C overnight, then washed twice for 5 min each with 0.2 M wash buffer (0.2 M Na2HPO4 pH 7.2, 1 mM EDTA, and 2% SDS) at room temperature and once for 10 min with 0.1 M wash buffer at 42°C. Relative telomere-repeat signals were determined by Typhoon 9410 Imager and ImageQuant TL software (GE Healthcare). Signal intensity represents the percentage decrease of average telomere signals relative to dox (-) or PP control.

TIF assay and RNA FISH

Indirect immunofluorescence (IF) combined with fluorescence *in situ* hybridization (FISH) analysis for telomere dysfunction was performed as described [4] with some modifications. Briefly, cells grown on coverslips were fixed for 15 min in 2% paraformaldehyde/1x PBS at RT, followed by permeabilization for 10 min in 1x PBS/0.5% Triton X-100 at RT. After washing with 1x PBS, cells were incubated for 60 min in blocking solution (0.5% BSA, 0.2% fish gelatin, 0.1% Triton X-100, 1 mM EDTA in 1x PBS) before immuno-staining. Primary antibodies were prepared in blocking solution at following dilutions: anti-53BP1 (1:500, IHC-00001; Bethyl Laboratories) and γH2AX (1:2,000, A300-081A; Bethyl Laboratories). After IF, cells were fixed in 4% paraformaldehyde in 1x PBS for 10 min, washed in 1x PBS, dehydrated in ethanol series (70%, 95%, 100%), and air-dried. Coverslips were denatured for 5 min at 80-85°C in hybridization mix [70% formamide, 10 mM Tris-HCl, pH 7.2, and 0.5% blocking solution (Roche)] containing telomeric PNA-Cy3-$(CCCTAA)_3$ probe, and hybridization was continued for 2 hrs at room temperature in the dark moisturized chambers. Coverslips were washed twice for 15 min each with 70% formamide, 10 mM Tris-HCl (pH 7.2), and 0.1% BSA, and followed by three washes for 5 min each with 0.15 M NaCl, 0.1 M Tris-HCl (pH 7.2), and 0.08% Tween-20. Nuclei were counterstained with 0.1 μg/ml DAPI in blocking solution and coverslips mounted with VectorShield (Vector Laboratories). RNA FISH was performed as previously described [1]. Images were captured with a 100x lens on a Nikon E600 Upright microscope (Nikon

Instruments, Inc., Melville, NY) with ImagePro Plus software (Media Cybernetics, Silver Spring, MD) for image processing. Cells with $\geq$ 5 53BP1/γH2AX foci co-localizing with telomere DNA foci were scored as positive for TIF. The quantification was generated from at least three independent Immuno-FISH experiments.

Metaphase Telomere FISH

FISH analysis on metaphase spreads was performed as previously described [5]. Briefly, LOX cells were treated with indicated chemicals (2 μM) for 3 days in a 37˚C CO_2 incubator. LOX TRF2ΔB cells were cultured in the absence (-) or presence (+) of 1 μg/ml doxycycline for 20 days in 37˚C incubator. Colcemid (0.1 mg/ml; GIBCO) was then added to cells and incubated for 4 hrs to accumulate mitotic cells. Cells were collected by trypsinization and suspended in 75 mM KCl hypotonic solution at 37˚C for 25 min before fixation in fresh 3:1 methanol/acetic acid for 4-5 times. Fixed cells were dropped onto cold, wet glass microscope slides and allowed to dry slowly in a humid environment. Metaphase chromosome spreads were fixed in 4% paraformaldehyde in 1x PBS for 3 min, dehydrated in ethanol series [70%, 95%,100% (vol/vol)], and air-dried. Slides were denatured for 5 min at 81 °C in hybridization mix [70% (vol/vol) formamide, 10 mM Tris·HCl (pH 7.2), and 0.5% blocking solution (Roche)] containing telomeric PNA-Cy3-(CCCTAA)3 probe (PNA Bio). After denaturation, hybridization was continued for 2 h at room temperature in the dark. Slides were washed twice for 15 min with 70% (vol/vol) formamide, 10 mM Tris·HCl (pH 7.2), and 0.1% BSA, and then three times for 5 min each with 0.15 M NaCl, 0.1 M Tris·HCl (pH 7.2), and 0.08% Tween-20. Nuclei were counterstained with 0.1 µg/mL DAPI in 1x PBS and slides were mounted with VectorShield (Vector Laboratories). Images were taken with a 100x lens on a Nikon E600 Upright microscope (Nikon Instruments) using ImagePro Plus software (Media Cybernetics) for image processing. Statistical analysis was performed using two-tailed Student t Test.

Nascent RNA Capture

Nascent RNA analysis was performed as described previously [2] by the use of Click-iT Nascent RNA capture kit (Life Technologies) with some modifications. Briefly, LOX cells were treated with 2 μM of chemicals, or LOX TRF2ΔB cells were induced with 1 μg/ml of doxycycline for 48 hrs, and then treated cells were pulse labeled 0.2 mM EU for additional 4 hrs. Total RNA was prepared and biotinylated by Click reaction. Nascent RNA was purified by using Streptavidin T1 magnetic beads and was subject to cDNA synthesis using SuperScript VILO cDNA synthesis kit (Life Technologies). Relative Nascent RNA expression was determined using ΔCT methods relative to control gene *U1* or *Actin*. Primer sequences used in qPCR analysis of 10q, 13q, XYq, and 18q TERRA were described previously [2].

Reference (Supplmentary Methods)

1 Deng, Z., Norseen, J., Wiedmer, A., Riethman, H. & Lieberman, P. M. TERRA RNA binding to TRF2 facilitates heterochromatin formation and ORC recruitment at telomeres. *Mol Cell* **35**, 403-413, doi:10.1016/j.molcel.2009.06.025 (2009).

2 Deng, Z. *et al.* HSV-1 remodels host telomeres to facilitate viral replication. *Cell Rep* **9**, 2263-2278, doi:10.1016/j.celrep.2014.11.019 (2014).

3 Deng, Z. *et al.* A role for CTCF and cohesin in subtelomere chromatin organization, TERRA transcription, and telomere end protection. *EMBO J* **31**, 4165-4178, doi:10.1038/emboj.2012.266 (2012).

4 Li, F. *et al.* ATRX loss induces telomere dysfunction and necessitates induction of alternative lengthening of telomeres during human cell immortalization. *EMBO J* **38**, e96659, doi:10.15252/embj.201796659 (2019).

5 Deng, Z., Dheekollu, J., Broccoli, D., Dutta, A. & Lieberman, P. M. The origin recognition complex localizes to telomere repeats and prevents telomere-circle formation. *Curr Biol* **17**, 1989-1995, doi:10.1016/j.cub.2007.10.054 (2007).

Supplemental Figure Legends

Figure S1. Different monovalent cations affect the G-quadruplex features of TERRA

CD spectroscopy curves showed TERRA displayed a typical parallel G-quadruplex structure in 150mM KCl (red) and the peak value decreased in 150mM LiCl (orange), while the control RNA $(CACUGA)_4$ displayed features of random RNA in both 150mM KCl (dark blue) and LiCl (light blue).

Figure S2. G-quadruplex structure is required for G-quadruplex-interacting compounds bound to telomeric nucleic acid repeats.

FP assays on compounds PP, NMM, BRACO-19 with:

A. TERRA antisense.

B. TeloDNA antisense.

C. TRF2 GAR peptide incubated with TERRA antisense.

D. TRF2 GAR peptide incubated with TeloDNA antisense.

All resulting FP values were measured from 3 independent experiments and plotted as a function of compound concentration using Prism 8.0.

Figure S3. Direct interaction between different ligands and telomeric nucleic acids.

FP assays on TERRA, antisense TERRA, TeloDNA and antisense TeloDNA with:

A. Pentacationic manganese (III) porphyrin 1 (PM3P)

B. Phen-DC3

C. Acridinium methosulfate (RHPS4)

D. Pyridostatin (PDS)

E. Summary of the direct interaction between telomeric nucleic acids and compounds using FP assay

All resulting FP values were measured from 3 independent experiments and plotted as a function of compound concentration using Prism 8.0. EC_{50} were presented in each graph.

Figure S4. G4 EBNA1 mRNA directly interacts with some G4-interacting compounds

FP assays indicates high binding affinity of G4 EBNA1 mRNA with pyridostatin and BRACO-19, but not NMM and PP. All resulting FP values were measured from 3 independent experiments and plotted as a function of compound concentration using Prism 8.0. EC_{50} were presented in each graph.

Figure S5. Homogeneous Time Resolved Fluorescence (HTRF) assay measures the direct interaction between TRF2 peptide and telomeric nucleic acids and the inhibition of these interactions by G4-interacting compounds.

A. Diagram of HTRF assay for interaction between FITC labeled peptide with biotin labeled nucleic acids with or without compounds disruption.

B. Direct interaction between TRF2 peptide and four nucleic acids measured by HTRF assay. K_d value for TRF2 interaction with TERRA G4 or TeloDNA G4 were presented in the graph.

C. HTRF assay measures the disruption of TERRA (G4 RNA) and TRF2 peptide interaction by compounds (BRACO-19, NMM and PP).

D. HTRF assay measures the disruption of TeloDNA (G4 DNA) and TRF2 peptide interaction by compounds (BRACO-19, NMM and PP).

All resulting HTRF values were measured from 3 independent experiments and plotted as a function of compound concentration using Prism 8.0 and calculation of IC_{50}.

Figure S6. Characterization of TERRA and telomere DNA after treatment with G4 interacting drugs.

A. DNA ChIP assay with TRF2 or IgG from LOX cells treated with 2 μM PP, NMM, or BRACO-19 for 2 days, and assayed for telomeric DNA with ^{32}P-labeled TelG probe or Alu control probe by Dot blot.

B. Quantitation of Dot blot assays represented in panel A. ns- no statistical difference between drug treated samples, two-tailed student t-test.

C. LOX cells were mock treated or treated with 2 μM PP, NMM or BRACO-19 for 2 days. Total RNA was isolated and assayed by Dot blot using ^{32}P-labeled probes containing the $(CCCTAA)_4$, or 18S sequence. TERRA signals relative to 18S RNA signals and signals from mock treated samples were shown at the bottom.

D. U2OS cells were treated with 2 μM PP, NMM or BRACO-19 for 2 days, and assayed by Dot blot using ^{32}P-labeled probes containing the $(CCCTAA)_4$ or 18S sequence. TERRA signals relative to 18S RNA signals and signals from PP were shown at the bottom.

E. LOX cells were treated with 2 μM PP, NMM, BRACO-19, or PDS for 3 days. Total RNA was isolated and assayed by Dot blot using ^{32}P-labeled probes containing the $(CCCTAA)_4$ (for TERRA), or 18S sequence. 3 μg of RNA was used for each sample and RNase A treatment (+) was used to assess possible DNA contamination.

F. Quantification of at least three independent RNA Dot blot assays as represented in panel **E**. Values are the means and SD (error bars). Two-tailed t-test, ** = $p < 0.01$, *** = $p < 0.001$, relative to PP control.

G. Telomere length analysis of LOX cells treated with 5 μM PP, NMM, BRACO-19, or 2 μM PDS for three days. Quantification of total telomere repeat signals for each sample as shown below each lane. Relative signals represent the telomeric signals relative to PP, which was arbitrarily set at 100.

H. LOX cells were treated with 2 μM PP, NMM, PDS, or solvent control (SC) for 2 days and then assayed by ChIP with BG4 antibody or IgG control. ChIP DNA was assayed by Dot blot probed for telomeric G4 DNA (TelC probe), as shown in right panel, and quantified as percentage of input for each treatment. Two-tailed t-test, * p-value <0.5 comparing NMM to PP.

Figure S7. Telomere damage foci induced by G4 interacting drugs.

A. LOX cells were mock treated or treated with 2 μM PP, NMM or BRACO-19 for 3 days, and assayed by IF FISH for TIF formation. 53BP1 foci are shown in green and telomere DNA foci are shown in red. The merged and DAPI counterstained images are shown in the right panels.

B. Quantification of IF FISH scoring for percentage of cells with ≥ 5 TIFs. The bar graph depicts the mean and SDs from three independent TIF assays (> 100 cells were scored). p-values calculated by two-tailed student t-test.

Figure S8. TRF2ΔB expression reduces TERRA levels in both LOX and U2OS cells.

A. LOX and LOX TRF2ΔB cells cultured in the absence (-) or presence (+) of doxycycline (1 μg/ml) for 2 days were assayed by dot blot using probes containing the $(CCCTAA)_4$, or 18S sequence. TERRA signals relative to 18S RNA signals and LOX Dox (-) were shown at the bottom.

B. TERRA RNA levels were assayed by dot blot during the course of doxycycline (1 μg/ml) induction in LOX TRF2ΔB cells, as indicated. TERRA signals relative to 18S RNA and Dox (-) signals are shown at the bottom.

C. Western blot of cell lysates of LOX TRF2ΔB during the time course of Dox induction, as shown in panel **B**. Myc-tagged TRF2ΔB, p53, p21, H3K9me3, and H3 in the absence (-) or presence (+) of Dox at indicated time points are shown.

D. RNA dot blot was used to assay TERRA levels in U2OS cells stably expressing Dox inducible TRF2ΔB. TERRA signals relative to signals from 18S RNA and Dox (-) at day 3 are shown at the bottom.

E. Western blot of cell lysates of U2OS TRF2ΔB cells. Myc-tagged TRF2ΔB and Actin in the absence (-) or presence (+) of Dox at day 6 are shown.

F. Total RNA (6 μg) isolated from U2OS TRF2ΔB cells cultured in the absence (-) or presence (+) of doxycycline (1 μg/ml) for indicated times were analyzed by Northern blot. RNA was detected with probe for TERRA, or 18S, as indicated. Ethidium Bromide (EtBr) staining of Northern gel is shown in bottom panel to indicate the stability of total RNA.

G. Representative telomere FISH analysis on metaphase spreads of LOX TRF2ΔB cells cultured for 20 days in the presence (+) or absence (-) of doxycycline. Stars (*) indicate signal free telomere ends and white arrows (>) indicate fragile telomere doublets. Magnified views of fragile telomeres indicate common telomere aberrations: normal metaphase telomeres (i); loss of telomeric signals (ii-iv); and fragile telomere doublets (v-vi). Scale bar, 10 μm.

Figure S9. Original images associated with figures.

Figure S1.

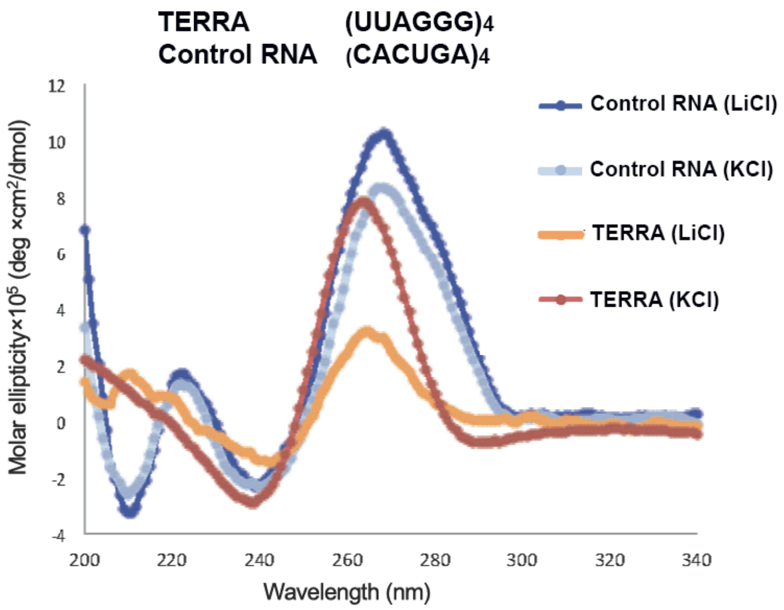
TERRA (UUAGGG)4
Control RNA (CACUGA)4
Molar ellipticity×10^5 (deg ×cm²/dmol)
12
10
8
6
4
2
0
-2
-4
200
220
240
260
280
300
320
340
Wavelength (nm)
Control RNA (LiCl)
Control RNA (KCl)
TERRA (LiCl)
TERRA (KCl)

Figure S2.

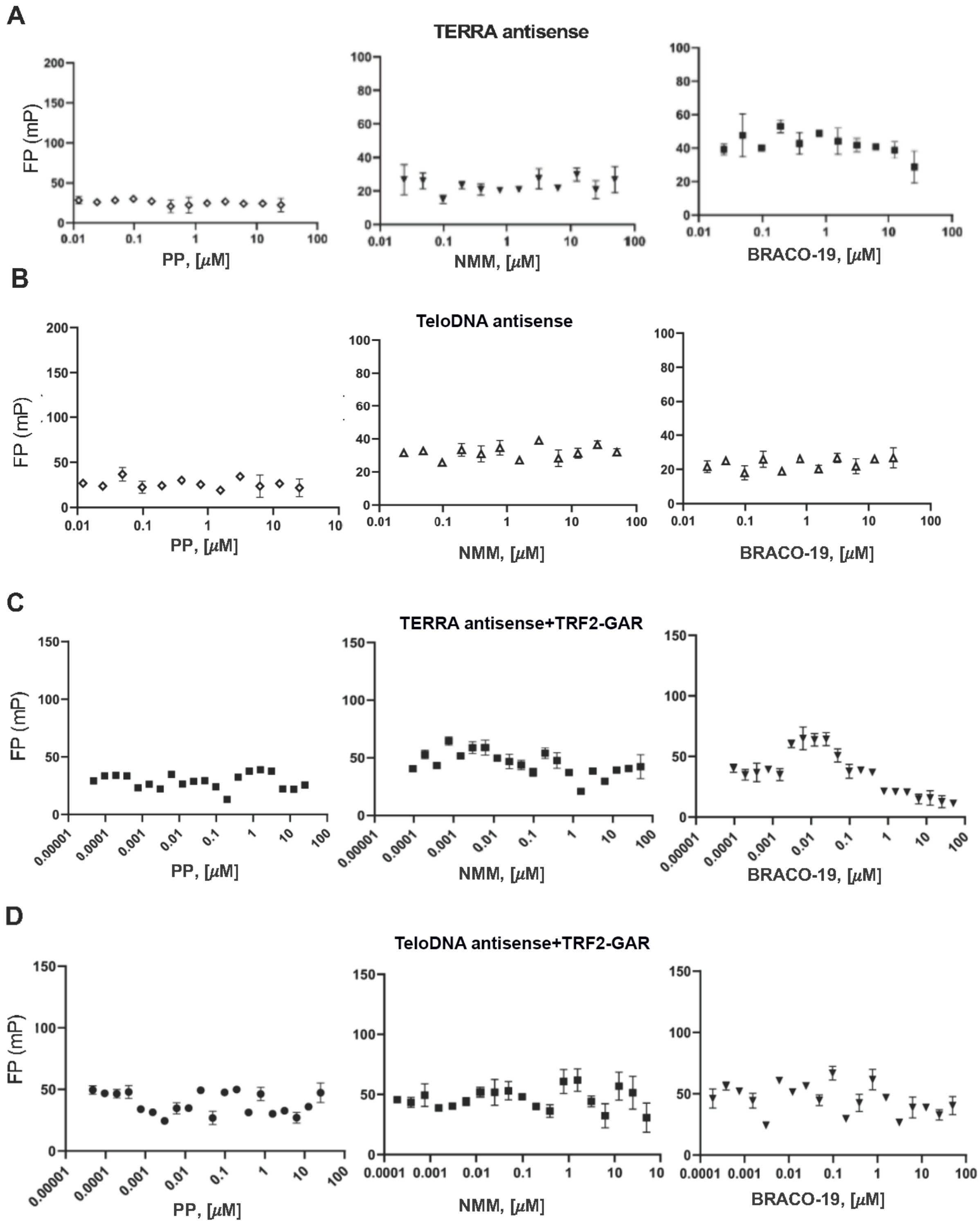
A
TERRA antisense
FP (mP)
PP, [μM]
NMM, [μM]
BRACO-19, [μM]
B
TeloDNA antisense
FP (mP)
PP, [μM]
NMM, [μM]
BRACO-19, [μM]
C
TERRA antisense+TRF2-GAR
FP (mP)
PP, [μM]
NMM, [μM]
BRACO-19, [μM]
D
TeloDNA antisense+TRF2-GAR
FP (mP)
PP, [μM]
NMM, [μM]
BRACO-19, [μM]

Figure S3.

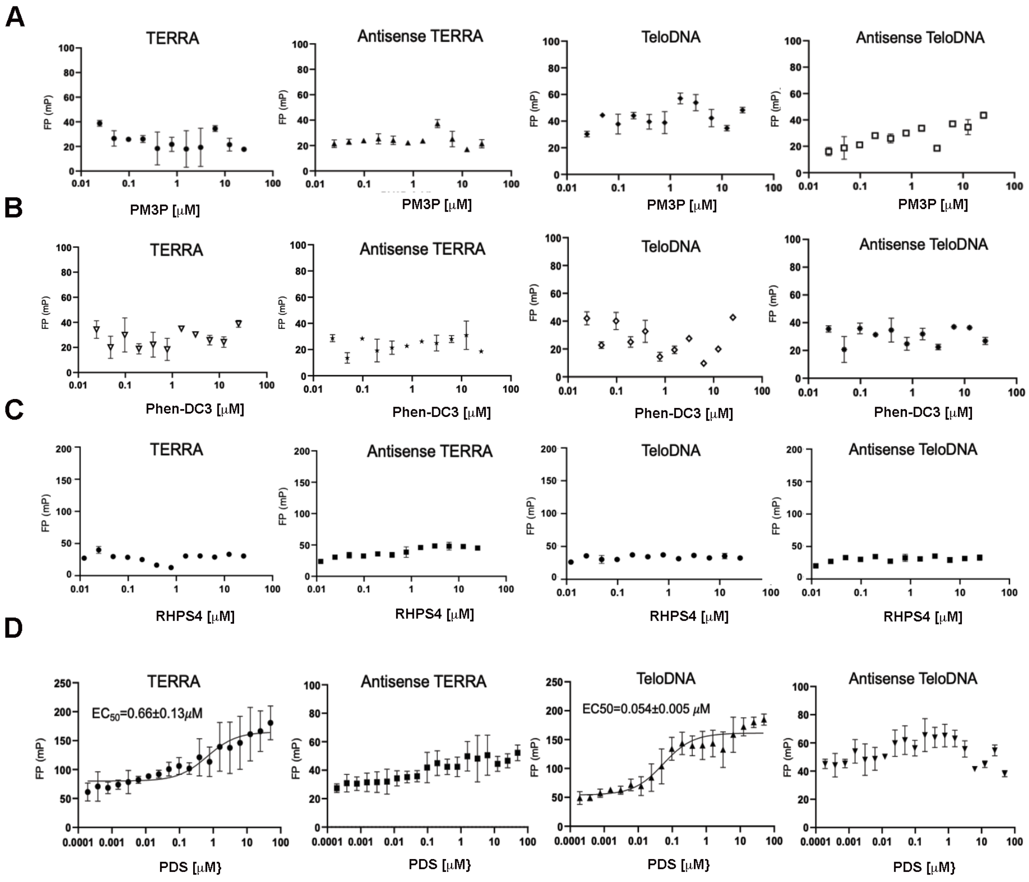

E

Summary of direct interaction between G4 nucleic acids and compounds

Ligands	TERRA	Antisense TERRA	TeloDNA	Antisense TeloDNA
PM3P	No binding	No binding	No binding	No binding
Phen-DC3	No binding	No binding	No binding	No binding
RHPS4	No binding	No binding	No binding	No binding
PDS	0.66±0.13μM	No binding	0.054±0.005μM	No binding

Figure S4.

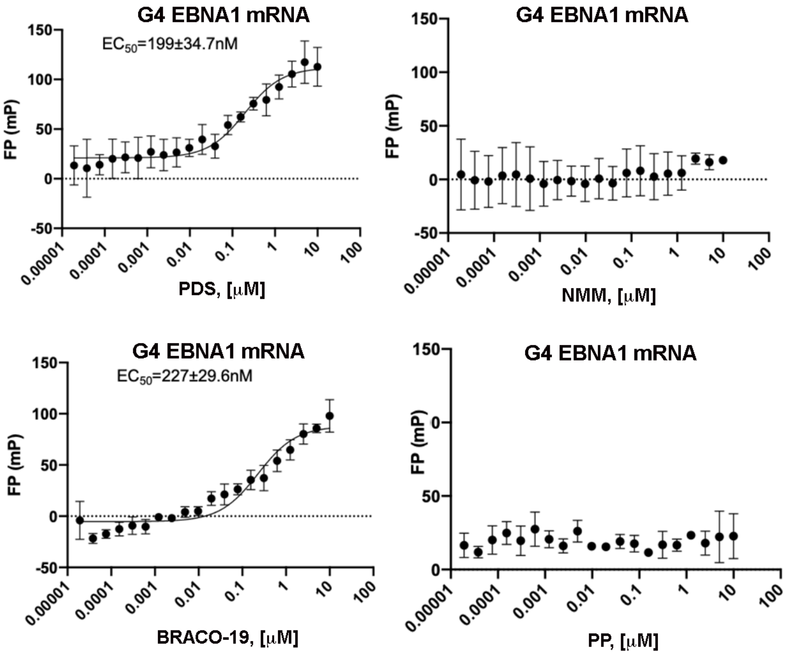
G4 EBNA1 mRNA
EC_{50}=199±34.7nM
FP (mP)
PDS, [μM]
G4 EBNA1 mRNA
FP (mP)
NMM, [μM]
G4 EBNA1 mRNA
EC_{50}=227±29.6nM
FP (mP)
BRACO-19, [μM]
G4 EBNA1 mRNA
FP (mP)
PP, [μM]

Figure S5.

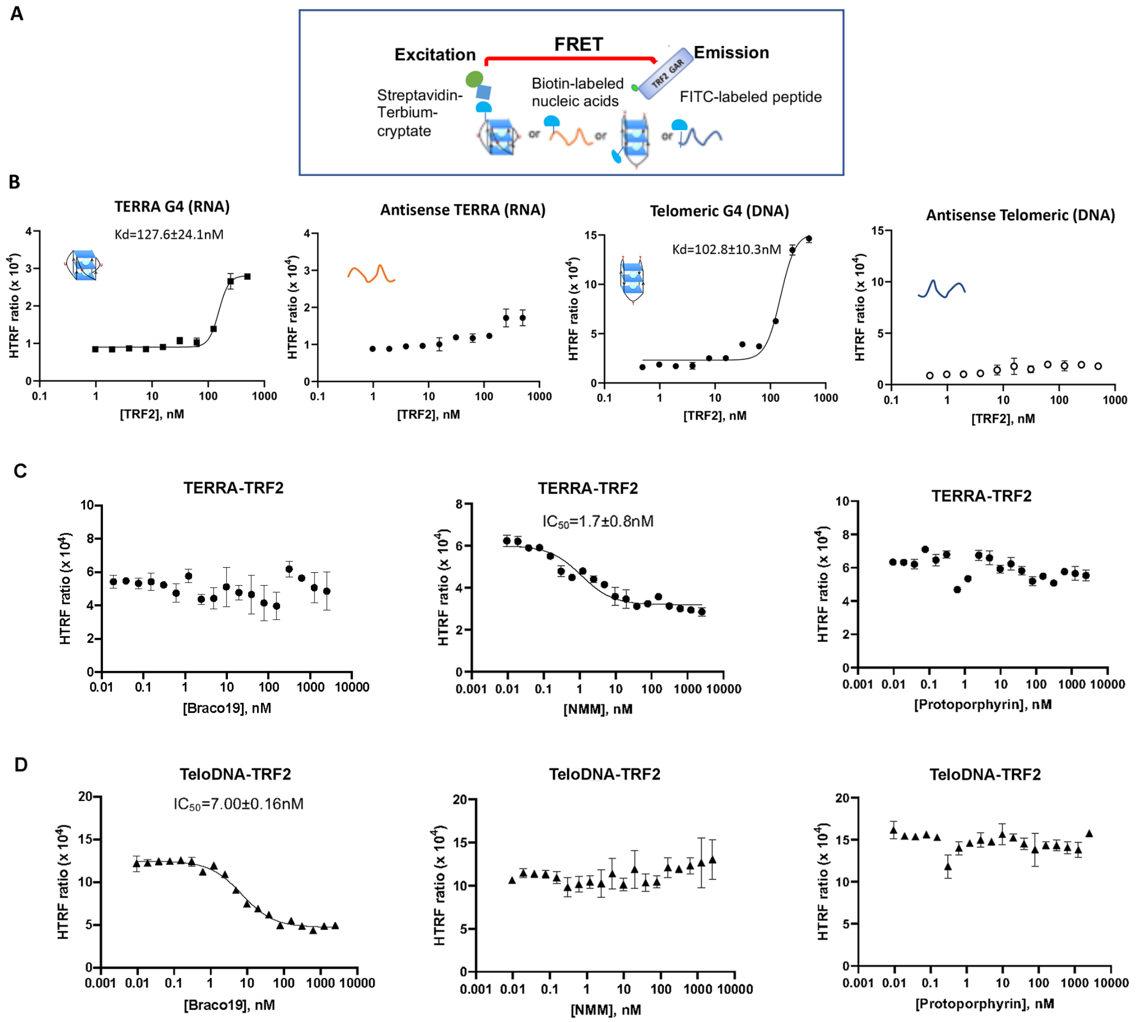
A
Excitation
FRET
Emission
Streptavidin-
Terbium-
cryptate
Biotin-labeled
nucleic acids
FITC-labeled peptide
TRF2 GAR
B
TERRA G4 (RNA)
Kd=127.6±24.1nM
Antisense TERRA (RNA)
Telomeric G4 (DNA)
Kd=102.8±10.3nM
Antisense Telomeric (DNA)
HTRF ratio (x 10⁴)
[TRF2], nM
C
TERRA-TRF2
IC50=1.7±0.8nM
[Braco19], nM
[NMM], nM
[Protoporphyrin], nM
D
TeloDNA-TRF2
IC50=7.00±0.16nM
[Braco19], nM
[NMM], nM
[Protoporphyrin], nM

Figure S6.

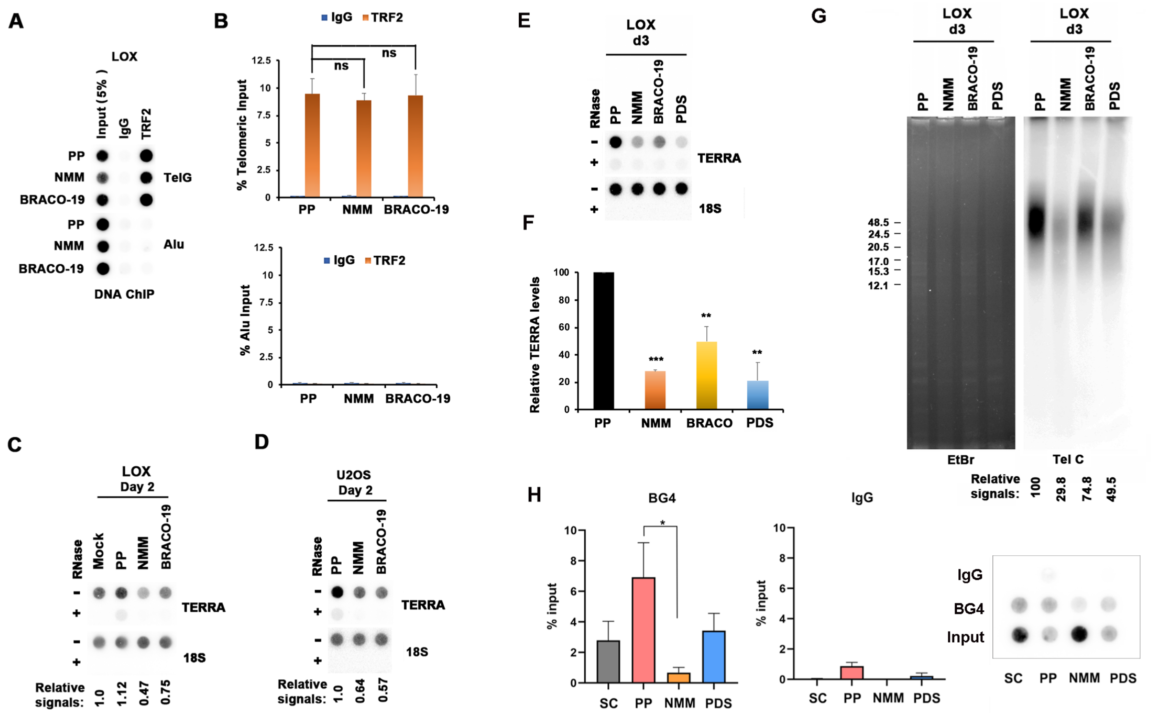
A
LOX
Input (5%)
IgG
TRF2
PP
NMM
BRACO-19
TelG
PP
NMM
BRACO-19
Alu
DNA ChIP
B
IgG TRF2
ns
ns
% Telomeric Input
PP NMM BRACO-19
% Alu Input
PP NMM BRACO-19
C
LOX
Day 2
RNase
Mock
PP
NMM
BRACO-19
TERRA
18S
Relative signals: 1.0 1.12 0.47 0.75
D
U2OS
Day 2
RNase
PP
NMM
BRACO-19
TERRA
18S
Relative signals: 1.0 0.64 0.57
E
LOX
d3
RNase
PP
NMM
BRACO-19
PDS
TERRA
18S
F
Relative TERRA levels
PP NMM BRACO PDS
G
LOX
d3
PP NMM BRACO-19 PDS
48.5
24.5
20.5
17.0
15.3
12.1
EtBr
Tel C
Relative signals: 100 29.8 74.8 49.5
H
BG4
IgG
% input
SC PP NMM PDS
IgG
BG4
Input
SC PP NMM PDS

Figure S7.

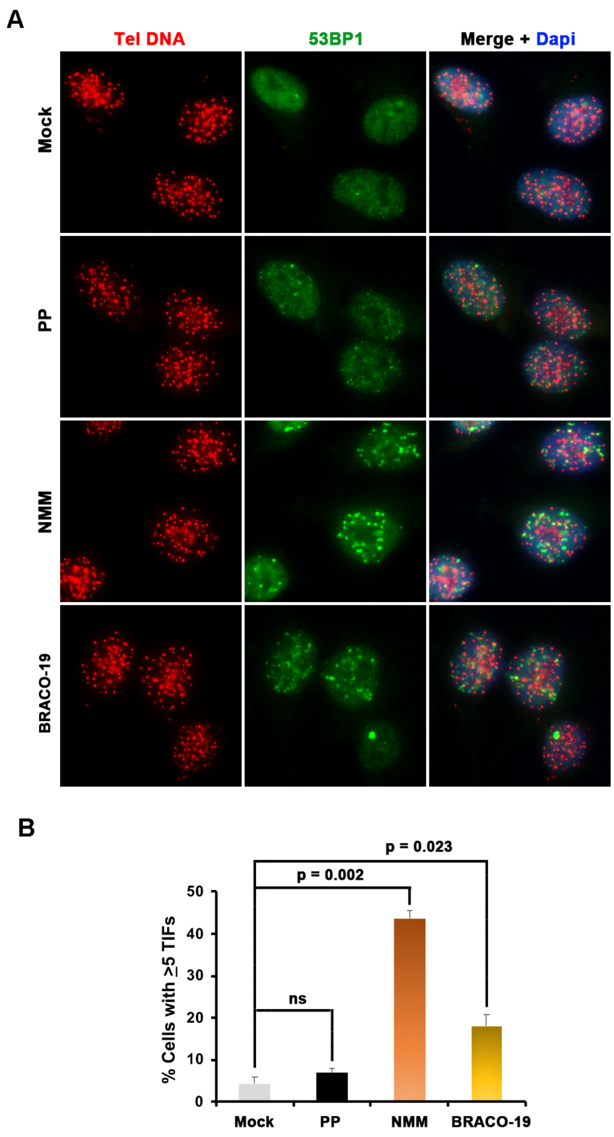
A
Tel DNA
53BP1
Merge + Dapi
Mock
PP
NMM
BRACO-19
B
p = 0.023
p = 0.002
ns
% Cells with ≥5 TIFs
50
40
30
20
10
0
Mock
PP
NMM
BRACO-19

Figure S8.

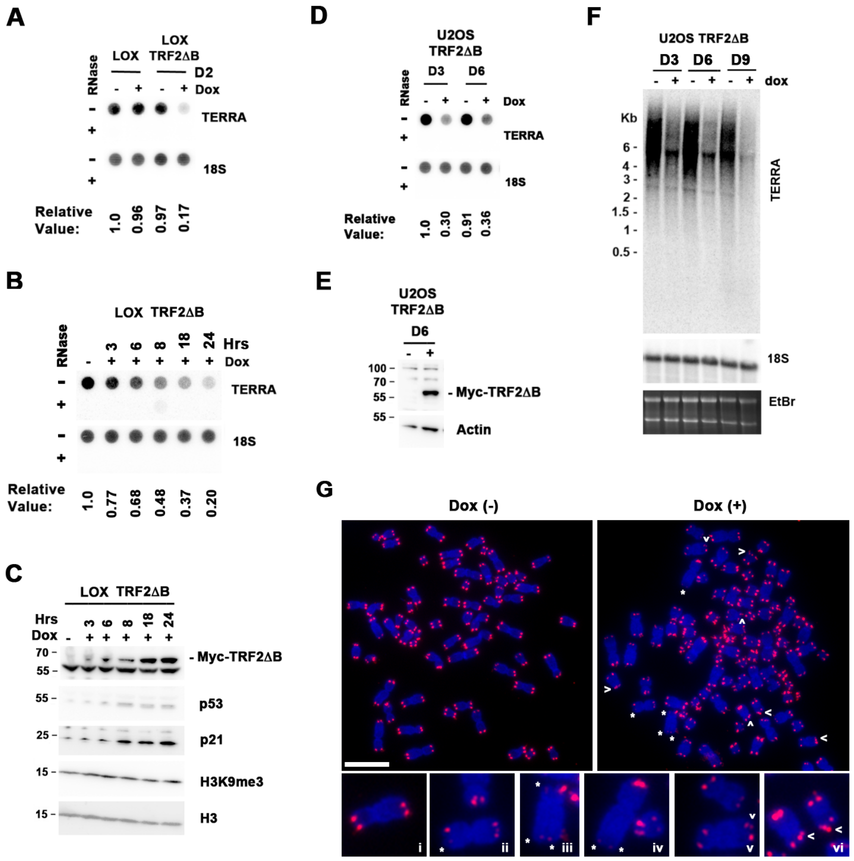
A
LOX
LOX TRF2ΔB
D2
RNase
Dox
TERRA
18S
Relative Value:
1.0 0.96 0.97 0.17
B
LOX TRF2ΔB
Hrs
Dox
TERRA
18S
Relative Value:
1.0 0.77 0.68 0.48 0.37 0.20
C
LOX TRF2ΔB
Hrs
Dox
Myc-TRF2ΔB
p53
p21
H3K9me3
H3
D
U2OS TRF2ΔB
D3 D6
Dox
TERRA
18S
Relative Value:
1.0 0.30 0.91 0.36
E
U2OS TRF2ΔB
D6
Myc-TRF2ΔB
Actin
F
U2OS TRF2ΔB
D3 D6 D9
dox
Kb
TERRA
18S
EtBr
G
Dox (-)
Dox (+)

Figure S9.

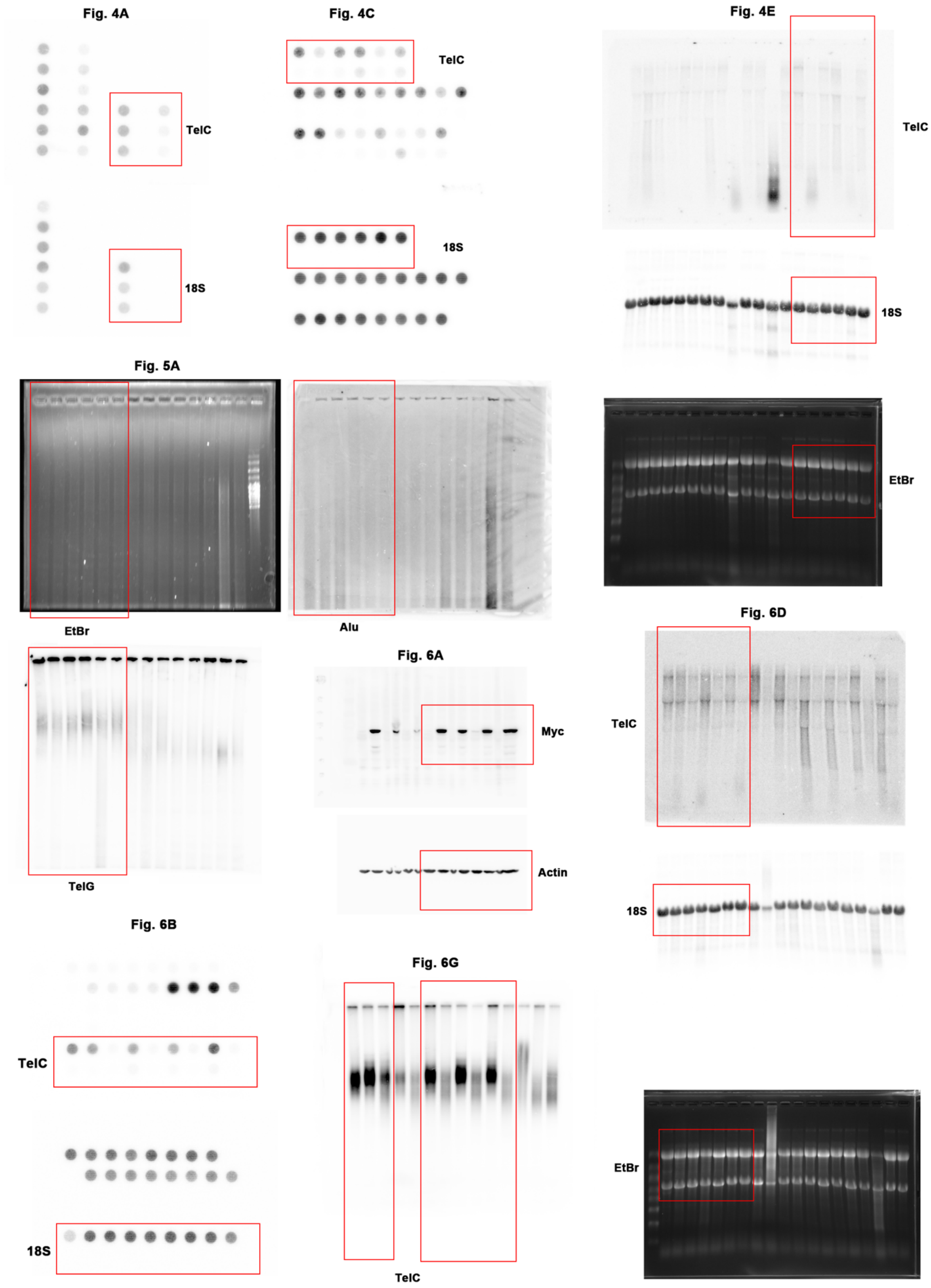
Fig. 4A
TelC
18S
Fig. 4C
TelC
18S
Fig. 4E
TelC
18S
EtBr
Fig. 5A
EtBr
Alu
TelG
Fig. 6A
Myc
Actin
Fig. 6D
TelC
18S
Fig. 6B
TelC
18S
Fig. 6G
TelC
EtBr

Figure S9 (continued).

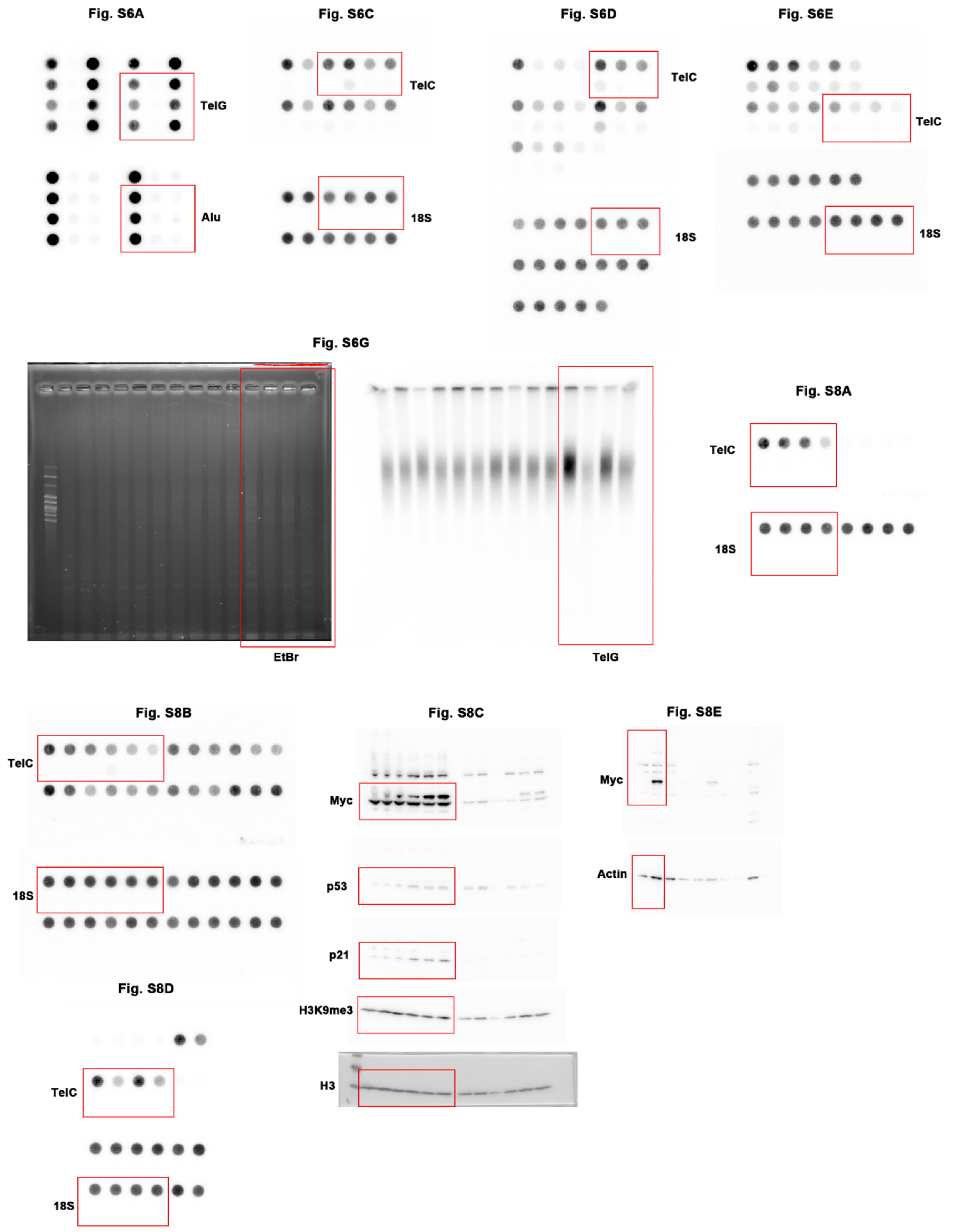

Figure S9 (continued).

Fig. S8F

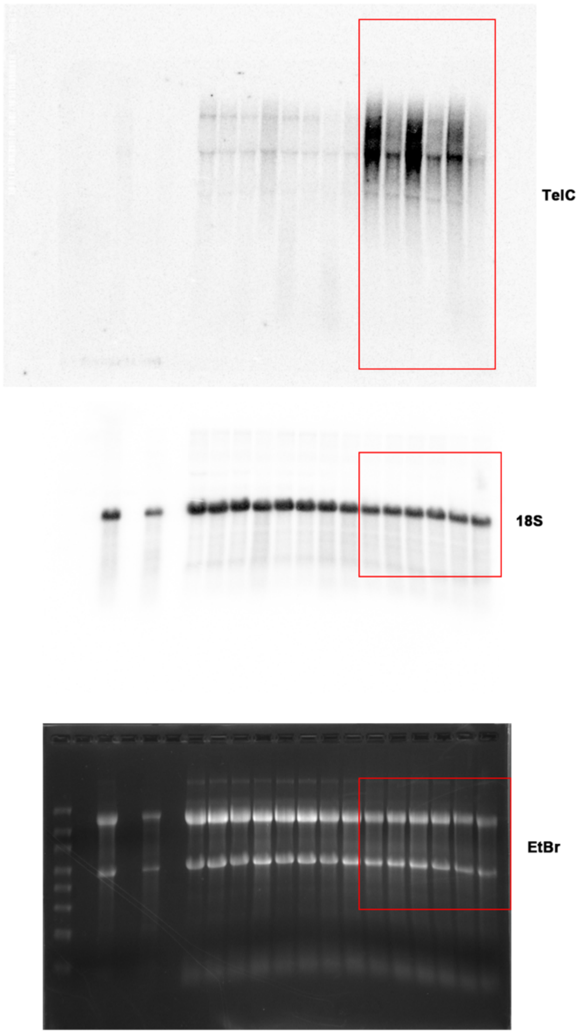

Full length TRF2 protein used in Fig. 2A-B

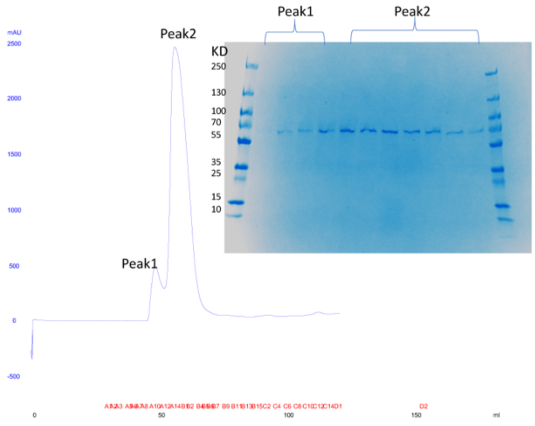

Supplement: Supplementary file 1 — Supplementary Information. [file 41598_2021_82406_MOESM1_ESM.pdf]
